# Preventable cancer cases and deaths attributable to tobacco smoking in Korea from 2015 to 2030

**DOI:** 10.4178/epih.e2025008

**Published:** 2025-02-27

**Authors:** Soseul Sung, Jihye An, Jeehi Jung, Hyeon Sook Lee, Sungji Moon, Inah Kim, Jung Eun Lee, Aesun Shin, Sun Ha Jee, Sun-Seog Kweon, Min-Ho Shin, Sangmin Park, Seungho Ryu, Sun Young Yang, Seung Ho Choi, Jeongseon Kim, Sang-Wook Yi, Yoon-Jung Choi, Youjin Hong, Sangjun Lee, Woojin Lim, Kyungsik Kim, Daehee Kang, Keun-Young Yoo, Sohee Park, Jeong-Soo Im, Hong Gwan Seo, Hai-Rim Shin, Kwang-Pil Ko, Sue K. Park

**Affiliations:** 1 Department of Preventive Medicine, Seoul National University College of Medicine, Seoul, Korea; 2 Department of Biomedical Sciences, Seoul National University Graduate School, Seoul, Korea; 3 Cancer Research Institute, Seoul National University, Seoul, Korea; 4 Department of Epidemic Intelligence Service, Incheon Communicable Diseases Center, Incheon, Korea; 5 Department of Biomedicine & Health Science, The Catholic University of Korea, Seoul, Korea; 6 Incheon Public Health Policy Institute, Incheon, Korea; 7 Interdisciplinary Program in Cancer Biology, Seoul National University College of Medicine, Seoul, Korea; 8 Department of Occupational and Environmental Medicine, Hanyang University College of Medicine, Seoul, Korea; 9 Department of Food and Nutrition, Seoul National University, Seoul, Korea; 10 Integrated Major in Innovative Medical Science, Seoul National University Graduate School, Seoul, Korea; 11 Department of Epidemiology and Health Promotion, Institute for Health Promotion, Graduate School of Public Health, Yonsei University, Seoul, Korea; 12 Department of Preventive Medicine, Chonnam National University Medical School, Hwasun, Korea; 13 Department of Family Medicine, Seoul National University Hospital, Seoul, Korea; 14 Department of Occupational and Environmental Medicine, Kangbuk Samsung Hospital, Sungkyunkwan University School of Medicine, Seoul, Korea; 15 Department of Internal Medicine, Healthcare Research Institute, Seoul National University Hospital Healthcare System Gangnam Center, Seoul, Korea; 16 Graduate School of Cancer Science and Policy, National Cancer Center, Goyang, Korea; 17 Department of Preventive Medicine and Public Health, Catholic Kwandong University College of Medicine, Gangneung, Korea; 18 Veterans Health Service Medical Center, Seoul, Korea; 19 Division of Cancer Registration and Surveillance, National Cancer Center, Goyang, Korea; 20 Division of Healthy Environments and Population, Western Pacific Regional Office, World Health Organization, Manila, Philippines; 21 Clinical Preventive Medicine Center, Seoul National University Bundang Hospital, Seongnam, Korea

**Keywords:** Neoplasms, Smoking, Population attributable fraction, Epidemiology, Korea

## Abstract

**OBJECTIVES:**

Tobacco smoking is a major public health concern worldwide. This study aimed to assess its impact on cancer incidence and mortality by estimating the population attributable fraction (PAF) in the Korean population for 2015 and 2020 and by projecting future trends until 2030.

**METHODS:**

The Korean relative risk (RR) was calculated via a meta–analysis of RRs for individual cancers attributed to tobacco smoking, based on primary data analysis from the Korean Cohort Consortium. The PAF was estimated using the Levin formula with past and current prevalence rates and the number of cancer cases and deaths, assuming a 15-year latency period.

**RESULTS:**

The proportions of cancer cases and deaths in Korea attributable to tobacco smoking were similar to those calculated using Asian and global RRs for both male and female. In 2015 and 2020, tobacco smoking contributed to 14.32% and 13.17% of cancer cases and 21.70% and 20.69% of cancer deaths in adults, respectively. Among Koreans, smoking was responsible for 25.83% of new cancer cases in male in 2015, 23.49% in male in 2020, 1.46% in female in 2015, and 1.68% in female in 2020. In both years, smoking impacted mortality more strongly than incidence in Korean male and female (incidence in male: 25.83% and 23.49%; mortality in male: 32.09% and 30.41%; incidence in female: 1.46% and 1.68%; and mortality in female: 4.70% and 4.96%, respectively).

**CONCLUSIONS:**

Tobacco smoking causes cancers and deaths in Korea, however, it is preventable. Effective control policies that consider trends and vulnerabilities among female are required.

## GRAPHICAL ABSTRACT

[Fig f5-epih-47-e2025008]

## Key Message

In Korea, tobacco smoking accounted for 14.32% of incident cancers and 21.70% of cancer deaths in 2015, and 13.17% of incidence and 20.69% of mortality in 2020. The burden was much greater in male than in female in both years (male: incidence 25.83% in 2015 and 23.49% in 2020; mortality 32.09% in 2015 and 30.41% in 2020; female: incidence 1.46% in 2015 and 1.68% in 2020; mortality 4.70% in 2015 and 4.96% in 2020). Smoking remains a preventable driver of substantial cancer incidence and mortality, calling for stronger control policies that also address emerging vulnerabilities among female.

## INTRODUCTION

Tobacco smoking is the leading cause of preventable disease worldwide, accounting for approximately 14% of global deaths in 2019. Of these deaths, 12% are attributed to direct smoking and 2% to exposure to secondhand smoke. In 2019, smoking resulted in 8 million premature deaths globally, with lung cancer being the primary cause [1]. Notably, the International Agency for Research on Cancer (IARC) has classified tobacco smoking as a Group 1 carcinogen due to sufficient evidence of its carcinogenicity in humans. Various cancers have been categorized as Group 1, including oral, pharyngeal, esophageal, gastric, colorectal, liver, pancreatic, laryngeal, lung, cervical, ovarian, renal, bladder, and hematological malignancies, as sufficient evidence is available linking them to smoking exposure [2].

In Korea, current smoking rates among adults older than 19 differ significantly between male and female. From 1998 to the most recent year available, male smoking rates have exhibited a decreasing trend (66.3% in 1998; 34.0% in 2020) due to anti-smoking campaigns, tobacco taxation policies, and altered social perceptions [3-5]. In contrast, rates among female have increased slightly, from 6.5% in 1998 to 7.5% in 2018 [3]. Notably, smoking rates have more than doubled among young female aged 20-29 years, from 5.1% in 1998 to 10.9% in 2018 [3]. Moreover, the age at which individuals start smoking has steadily decreased (for male, from 20.8 years in 1998 to 18.8 years in 2018; for female, from 29.4 years in 1998 to 23.5 years in 2018) [6].

Previous studies have calculated the population attributable fraction (PAF) by considering the prevalence and relative risks (RRs) of past and current smoking compared with never smoking. In contrast, the present study aimed to verify the PAF values using both Korean and global RRs, applying the same methodology. Instead of using categorical variables, the study calculated the RR per 10 pack-year increment and the smoking-attributable RR for all cancers.

## MATERIALS AND METHODS

Tobacco smoking exposure was categorized into 3 groups: non-smoking, past smoking, and current smoking. Additionally, pack-years—a measure of smoking exposure dose—was calculated as an alternative metric by determining the number of cigarette packs smoked per day and the duration of smoking in years. Pack-years were calculated as a continuous variable for both current and past smokers, while non-smokers were assigned a pack-year value of 0.

Past and current smoking rates, along with mean smoking pack-years, were derived from data obtained from the Korean National Health and Nutrition Examination Survey (KNHANES) for the years 1998, 2001, and 2004-2015. Considering a latency period of 15 years, we computed the smoking-attributable cancer PAF for Korea in 2015 and 2020, compared these values to those of other countries, and projected the cancer PAF for 2025 and 2030 to estimate future proportions of cancer incidence and mortality attributable to smoking. As smoking is a modifiable risk factor, the PAF can provide an estimate of the potential reduction in cancer cases if smoking were to cease. These calculations were specifically performed for adults aged 20 years and older [3]. To estimate the sex-specific prevalence rates for past and current smoking in the year 2000, a linear regression model was used. This model utilized sex-specific rates standardized by the 2000 mid-year population, which were gathered from the years 1998, 2001, 2005, and 2007-2020.

For this study, indirect smoking and electronic cigarettes were excluded from the exposure factors because their association with cancer risk could not be established based on Korean cohort studies. Similarly, chewing tobacco use, which is rarely reported in Korea, was also excluded from the exposure factors. Smoking-related cancers were defined based on the IARC Group 1 classification, which includes cancers with sufficient evidence of carcinogenicity due to tobacco smoking. These cancers include those of the mouth, pharynx, and larynx (International Classification of Diseases, 10th revision [ICD-10] codes C00-C14, C32); esophagus (ICD-10 code C15); stomach (ICD-10 code C16); colorectum (ICD-10 codes C18-C20); liver (ICD-10 code C22); pancreas (ICD-10 code C25); lung (ICD-10 codes C33-C34); cervix (ICD-10 code C53); ovary (ICD-10 code C56); kidney (ICD-10 codes C64-C66); and bladder (ICD-10 code C67). Cancers included in the calculation of RRs were those for which the IARC has determined sufficient evidence of tobacco smoking as a carcinogen in humans (Supplementary Material 1).

To calculate cancer-specific RRs, we conducted a systematic literature review to identify the association between smoking and cancer risk in Korean cohort studies. Such studies have reported the incidence of common cancers, such as those of the lung, stomach, and liver. In contrast, ovarian cancer has only been reported in multicenter case-control studies [7-10], and no studies have examined leukemia. To overcome these limitations, we calculated the RR for tobacco smoking on cancer risk using raw data analysis of cohort studies registered in the Korean Cohort Consortium [11]. We determined sex-specific RRs by cancer type using multivariable Cox proportional hazards models, adjusting for age (continuous), alcohol consumption, body mass index (continuous), and regular physical activity (yes or no). We then meta-analyzed the RRs using a random-effects model [12]. For sensitivity analysis, we conducted a meta-analysis using a systematic review of Asian and global cohort studies on the association between smoking (past or current) and cancer risk, with non-smoking used as the reference group. Another sensitivity analysis was performed by conducting a meta-analysis to calculate the overall cancer risk attributable to smoking using primary data analysis from Korean cohort studies. Additionally, we conducted a meta-analysis to assess the cancer risk associated with a 10 pack-year increase in smoking. We obtained information on cancer incidence and mortality in adults aged 20 years and older in 2015 and 2020 from national cancer registration data and cause-of-death statistics [13,14].

The PAF for specific cancers in relation to past and current smoking compared to non-smoking was calculated using the formula proposed by Levin. The 95% confidence intervals (CIs) for the PAF were calculated using Monte Carlo methods [15-18]. The calculation of the cancer PAF proceeded as follows. First, we calculated the number of attributable cancer cases or deaths (ACs), which represent the number of cancer cases or deaths attributable to smoking, for specific cancers. We then summed the ACs across specific cancers to obtain the overall number of cancer cases or deaths attributable to smoking. Finally, we divided the ACs by the associated total number of cancer cases or deaths to calculate the PAF for smoking-associated cancers [19].

A sensitivity analysis was conducted for the PAF using meta-analyzed RRs from Asian and global cohort studies (including systematic reviews and Korean RRs from raw-data analyses), Korean RRs per 10 pack-years, and Korean RRs for all cancers. All RRs were calculated based on cohort studies. To project the PAF trend through 2030, PAF values for 2025 and 2030 were calculated using smoking rates from 2005, 2010, and 2015. This calculation was performed under the assumption of a 15-year latency period and a consistent RR. The methods for estimating the expected population and the expected number of cancer cases and deaths in 2025 and 2030 have been described in previous research [20]. The PAFs for cancer in 2015 were compared to those in 2009, which were calculated using smoking rates from 1990 with a 19-year latency period [9,10].

### Ethics statement

This study was approved by the Institutional Review Board of Seoul National University Hospital (IRB No. C-1911-188-1084).

## RESULTS

The smoking rates in the year 2000 were 62.3% for male and 5.9% for female, while the past smoking rates were 21.1% for male and 3.3% for female. Over time, current smoking rates have declined in male, whereas they have increased in female (Supplementary Material 2).

Both past and current smoking were associated with an increased risk of most cancers compared with non-smoking, in both sexes and for both incidence and mortality. Generally, the RRs were higher in male than in female, and the RRs for cancer mortality exceeded those for cancer incidence. In male, for both incidence and deaths, current smoking posed the highest risk for lung cancer (incidence: RR, 4.75; 95% CI, 3.39 to 6.66; death: RR, 4.69; 95% CI, 3.38 to 6.51). In contrast, among female, for both incidence and deaths, current smoking was associated with the highest risk for laryngeal cancer (incidence: RR, 11.73; 95% CI, 8.28 to 16.60; death: RR, 20.26; 95% CI, 11.08 to 37.03). Furthermore, current smoking was associated with a substantially higher risk of esophageal cancer mortality in female (RR, 11.84; 95% CI, 2.42 to 57.98) than in male (RR, 1.94; 95% CI, 1.81 to 2.08) (Supplementary Materials 3-5).

Sensitivity analysis using pack-years showed that for every 10 pack-year increase, the risk of lung cancer incidence increased by 1.27-fold in male and 1.43-fold in female. The risk of lung cancer mortality was similar to that for incidence. Additionally, the RRs for past and current smoking for all cancer deaths (ICD-10 codes C00-C96; 1.32 and 1.98 for male; 1.34 and 1.68 for female, respectively) were higher than those for all cancer cases (Supplementary Material 6).

In 2020, tobacco smoking contributed to 13.17% of cancer incidence and 20.69% of cancer-related deaths among Korean adults aged 20 years and older. The fraction attributable to tobacco smoking was higher in male than in female (23.49 vs. 1.68% for cancer cases and 30.41 vs. 4.96% for cancer deaths). Among male, smoking accounted for 70.55% of lung cancer cases and 70.18% of lung cancer deaths, whereas among female, it accounted for 10.08% of lung cancer cases and 16.41% of lung cancer deaths (Table 1, Supplementary Materials 7 and 8).

In 2015, 21.69% of all cancer cases in male and 0.90% in female (ICD-10 codes C00-C96) were attributable to active smoking, based on the RR for all cancers. This corresponded to 24,675 male and 1,224 female among all cancer cases. Additionally, in 2015, 39.21% of male cancer deaths and 5.44% of female cancer deaths were attributed to smoking, representing 18,641 male and 1,419 female. Notably, the PAF for cancer based on pack-years of smoking (8.4%) was lower than the categorical PAF based on non-smoking, past smoking, and current smoking in 2015 (Table 2, Supplementary Materials 9 and 10).

**Figure 1.**
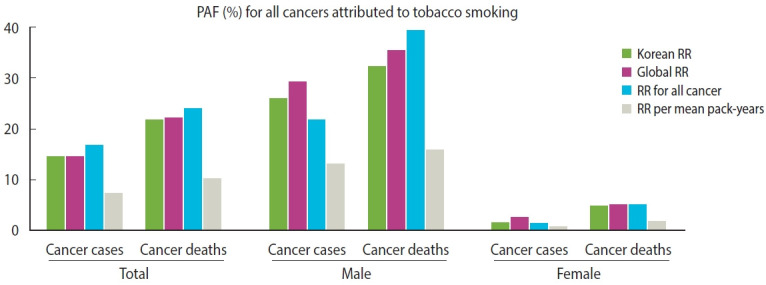
Comparison of population attributable fraction (PAF) in cancer attributed to tobacco smoking when using different relative risks (RRs).

The PAF values for cancer incidence calculated using Korean and Asian RRs were almost identical, whereas those calculated using global RRs (29.30% for male and 2.67% for female) were slightly higher than those obtained using Asian RRs (26.24% for male and 1.52% for female) and Korean RRs (25.83% for male and 1.46% for female). This pattern was consistent when assessing the proportion of smoking-attributable cancer deaths; the global PAFs were 35.40% for male and 5.12% for female, compared with 32.71% for male and 4.62% for female using Asian RRs and 32.09% for male and 4.70% for female using Korean RRs (Figure 1 and Table 2, Supplementary Material 11).

The number of cancer cases and deaths attributed to smoking increased from 2009 to 2015. The smoking-related PAF for cancer incidence rose from 11.90% in 2009 to 14.31% in 2015, while the PAF for cancer mortality decreased from 22.83% to 21.70%. Notably, the smoking-related PAF for cancer incidence increased more in male (from 20.94% in 2009 to 25.83% in 2015), primarily due to lower estimates for lung, colorectal, and stomach cancers in 2009 (lung cancer, 53.34% in 2009 vs. 72.27% in 2015; stomach cancer, 27.89 vs. 36.58%; and colorectal cancer, 1.50 vs. 17.81%) [9] (Supplementary Material 12).

**Figure 2.**
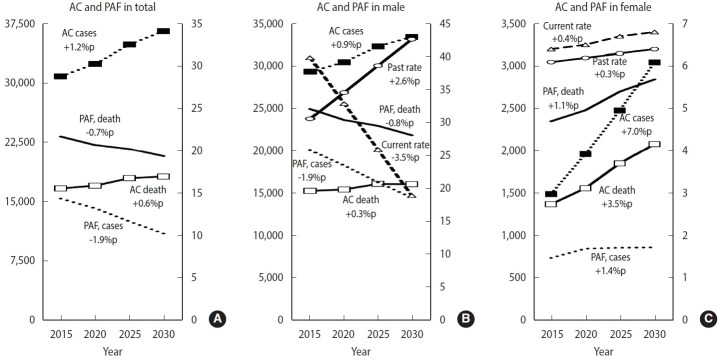
Changing trends of population attributable fraction (PAF) and attributable cancer cases and deaths (ACs) in cancer attributed to tobacco smoking in Korea, 2015 to 2030 (A) total, (B) male, and (C) female. Current and past rate means smoking prevalence rates. We calculated smoking prevalence and PAF values exclusively for male and female separately. %p, percentage point.

**Figure 3.**
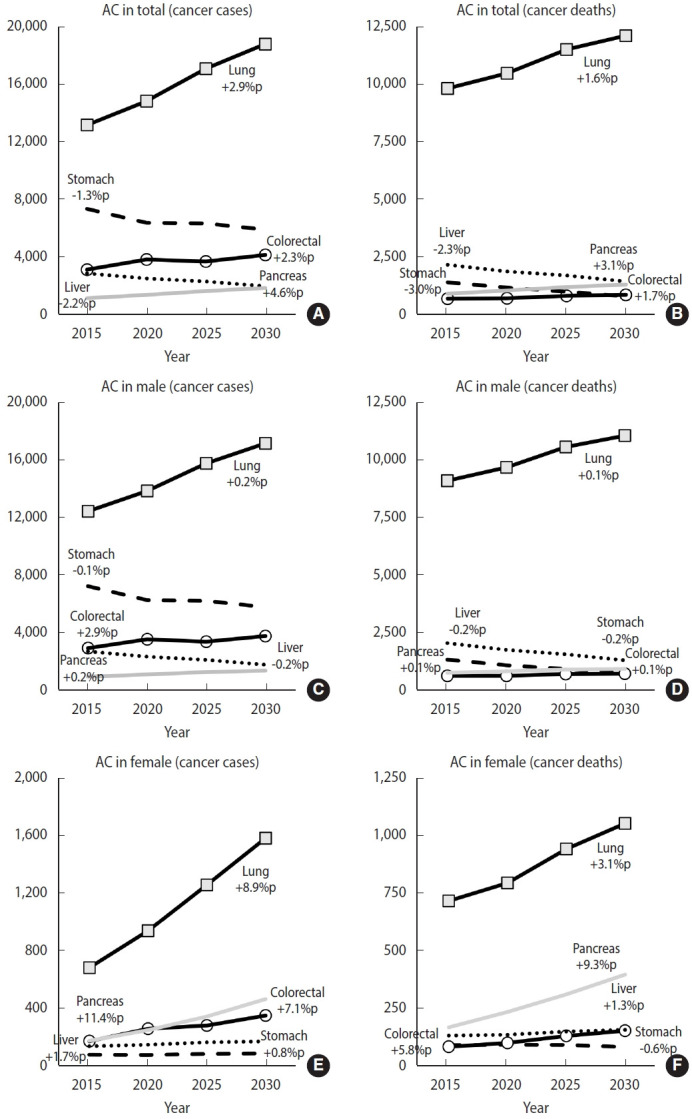
Changing trends of attributable cancer cases and deaths (ACs) in specific cancer attributed to tobacco smoking in Korea, 2015 to 2030. Attributable cancer cases in (A) total, (C) male and (E) female. Attributable cancer deaths in (B) total, (D) male, and (F) female. %p, percentage point.

The smoking-related PAF for cancer incidence and mortality in male is predicted to decline continuously from 2015 to 2030 (incidence, 25.83% in 2015 to 18.58% in 2030; mortality, 32.09% in 2015 to 28.09% in 2030). In contrast, for female, the smoking-related PAF for cancer is expected to be slightly higher in 2030 compared to 2015, with incidence rising from 1.46% to 1.71% and mortality increasing from 4.70% to 5.69%. An increasing trend in smoking-related PAF was observed for all cancers among female, with a greater rise in PAF for cancer mortality than for incidence (Figures 2 and 3).

## DISCUSSION

In 2015, tobacco smoking contributed to 14.32% of cancer cases among Koreans (25.83% in male; 1.46% in female) and accounted for 21.70% of cancer deaths (32.09% in male; 4.70% in female). Specifically, tobacco smoking was responsible for more than two-thirds of lung cancer incidence (72.27%) and mortality (71.92%) in male, indicating that a large proportion of lung cancer was due to smoking. Among female, tobacco smoking explained 42.01% of laryngeal cancer incidence and 56.71% of laryngeal cancer mortality, suggesting that smoking was a major contributor to laryngeal cancer in female (Table 2).

**Figure 4.**
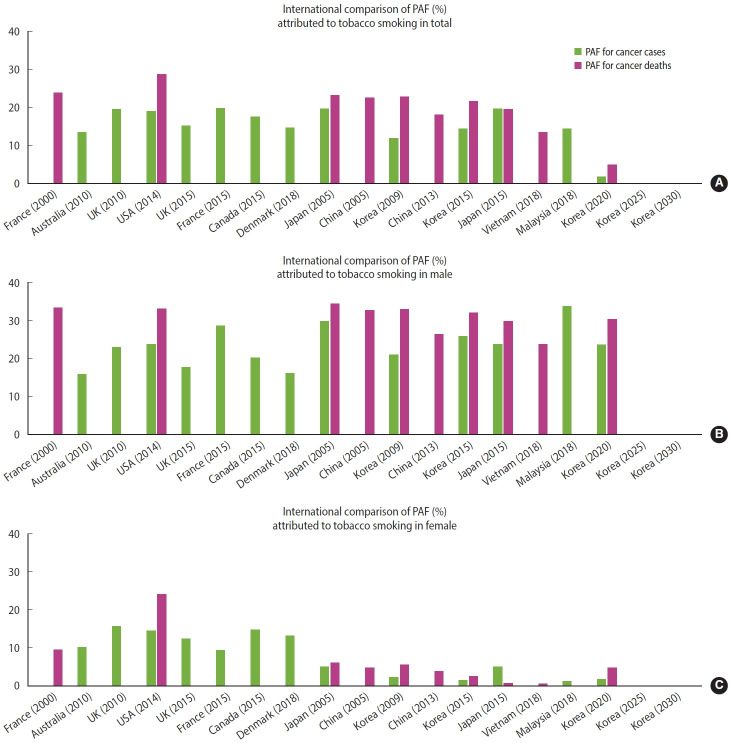
International comparison of population attributable fraction (PAF) attributed to tobacco smoking in (A) total, (B) male, and (C) female.

The distinct patterns of cancer PAF attributed to smoking have been observed in multiple countries. The contribution of smoking to cancer incidence varies from 16% to 35% for male and from 2% to 16% for female [21-28]. Moreover, the contribution of smoking among male is consistently high across countries. Furthermore, the cancer PAF attributable to smoking is higher for cancer deaths than for cancer incidence, a trend that has been observed in different nations [21-30]. This discrepancy is likely due to the additional impact of smoking on cancer mortality beyond its direct carcinogenic effects. Socioeconomic factors—such as poverty, limited access to healthcare, and health inequalities—contribute to a higher PAF for cancer deaths and may become more pronounced with increasing age [31]. Additionally, the relatively high mortality rates associated with smoking-related cancers, such as lung and stomach cancers, may also play a role (Figure 4).

Lung cancer exhibits the highest smoking-related PAF among all cancers, exceeding 80% in Western populations but displaying lower percentages in China (43% for deaths) and in our study (53.36% for incidence; 56.53% for deaths). These disparities stem from methodological differences in PAF calculation and risk estimation [22,27,28], as well as from ethnic variations in smoking-related cancer RRs [32,33]. Multiethnic cohort studies have highlighted these RR variations between Eastern and Western populations, which are influenced by genetic factors, such as *CYP2A6* variants, and socio-cultural behaviors affecting smoking habits [34-39].

The sensitivity analysis in this study indicates that cancer PAFs derived from average pack-years were lower than those based on categorical smoking statuses. Because pack-year data were available for only around 70% of cohorts, PAFs for past smokers were combined with the average pack-year data of current smokers. Notably, the PAFs for current smokers based on smoking quantity showed minimal variation, with values of 9.3% for male (≥20 years with ≥30 pack-years) and 0.9% for female (≥20 pack-years). This suggests a non-linear dose-response relationship. Moreover, in the overall population and among male, the global PAF estimates were higher than the Asian/Korean PAF estimates; however, no significant difference was observed between global and Korean PAF estimates among female [31,40-42].

Although global RRs were higher than Asian/Korean RRs among female, the lower smoking rates among Korean female (current smoking rate, 5.9%; past smoking rate, 3.3%) resulted in less pronounced differences in PAF estimates. Smoking prevalence among Asian female, including Korean female, is generally underestimated in survey data due to negative socio-cultural perceptions of smoking [43]. For example, when urinary cotinine levels were measured alongside self-reported smoking among Korean female, the actual smoking prevalence was estimated to be 1.3 times to 2.5 times higher than reported [43]. In contrast, among male, the ratio of survey-reported smoking prevalence to cotinine-based smoking prevalence was around 1.0 to 1.2, indicating close agreement; indeed, recent years have shown nearly identical survey-reported smoking prevalence among male [43].

In the present study, the PAF for smoking among female was calculated as 1.46% for cancer incidence and 4.70% for cancer mortality. However, when considering the actual smoking prevalence among female (assumed to be twice as high as reported), the PAF for cancer incidence among female was estimated at 2.51% and the PAF for cancer mortality at 7.51%. This suggests an underestimation of 1,238 cancer cases and 912 cancer deaths. Specifically, from 2015 to 2030, the smoking-related PAF for cancer is predicted to decrease among male but increase among female. Although several countries have implemented traditional tobacco control policies—such as tobacco taxation, smoking restrictions, and regulations on sales and advertising—sex-specific policies are rarely implemented [44]. Smoking among female is often perceived to be influenced by distinct socioeconomic factors, such as lower socioeconomic status and social isolation. However, the sex difference in lung cancer incidence is primarily explained by differences in smoking history. When smoking exposure is similar, sex differences in lung cancer risk are minimal [45]. This suggests that although socioeconomic factors may shape smoking behavior, discrepancies in the risk of lung cancer between male and female are largely driven by differences in smoking history.

This study analyzed data from the Korean Cohort Consortium, which encompasses all cohort studies conducted in Korea. Through meta-analysis, the cancer risks for Koreans were calculated using primary data analyses from each study investigator and national research institution. This systematic approach not only provides results for both cancer incidence and non-cancer outcomes but also overcomes limitations in statistical power. The study also employed a large-scale retrospective cohort dataset from the National Health Insurance Service to analyze the risk of smoking-related cancers, including rare ones. Additionally, for female, primary data analysis and meta-analysis of multiple cohorts were conducted to address limitations in previous research, associated with the limited exposure of this group to smoking. The study also provided results for cancer mortality risk.

When projecting the PAFs for 2025 and 2030 in this study, we assumed that the RRs associated with smoking remain constant over time. Although this approach is necessary to evaluate the impact of trends in smoking prevalence on PAF values, it does not account for potential changes in RRs due to advancements in healthcare or shifts in smoking behavior. Consequently, this assumption may limit the ability to fully capture the dynamic nature of smoking-related cancer risks over time. Additionally, changes in AC represent a key indicator of the absolute change in disease burden caused by smoking. Variations in AC reflect not only the influence of smoking prevalence but also actual changes in the number of cases attributable to smoking. This provides insight into how policy changes or shifts in health behaviors over time affect disease burden in a real-world context, highlighting the dynamic interplay between smoking patterns and cancer incidence.

Another limitation of this study is that we did not account for the impact of secondhand smoke exposure in the reference group of non-smokers when calculating the RRs. Secondhand smoke increases the risk of cancer and mortality even among non-smokers, potentially contributing to an underestimation of the RRs. The impact of secondhand smoke is a significant global health issue, and recent studies have reported increases in mortality and disability-adjusted life years attributable to secondhand smoke exposure [46]. This study acknowledges the importance of considering the effects of secondhand smoke, which should be addressed in future research.

In addition, the use of standardization in this study may not fully reflect recent changes in population structure or the impact of new risk factors. By standardizing based on the 2000 population, the study may not have accounted for demographic shifts or rapid changes in smoking behavior over time. Using actual population weights without standardization could provide a more accurate representation of these changes and their impact on smoking-related cancer PAFs.

A further limitation is the omission of increasingly popular novel tobacco products, such as e-cigarettes and heated tobacco products, which began to gain traction in 2011 and 2017. The rise in the use of these products could influence trends in smoking-related cancer PAFs, especially for female, and this should be considered in future research. As this study did not incorporate these emerging trends, the projections may underestimate the full impact of tobacco use on cancer incidence and mortality. In addition, this study did not account for the potential influence of competing risks, such as differences in disease burdens and mortality rates across countries, on smoking-attributable cancer risks. These competing risks can significantly influence the interpretation of cross-country comparisons. For instance, countries with high burdens of other diseases may experience lower cancer-specific mortality, thus impacting the relative contribution of smoking to cancer deaths. Recent studies have highlighted the role of competing risks in cancer epidemiology, emphasizing that differences in health systems, infectious disease prevalence, and other non-cancer mortality factors should be considered when making international comparisons [47,48].

Additionally, this study did not account for declines in daily smoking quantity among smokers. Although trends in smoking prevalence were considered, reductions in smoking intensity over time were not incorporated into the attributable fraction calculations. This omission may influence the accuracy of the findings, and future studies should consider the impact of changes in smoking intensity to provide a more comprehensive estimate of the disease burden attributable to smoking. Moreover, the study did not account for the full cumulative effect of smoking, including intensity over time and the risk reduction after cessation among ex-smokers. Although we calculated the RR and rate for ex-smokers separately, the impact of smoking intensity and the reduction in risk after cessation were not incorporated. Future studies should consider these factors to more accurately estimate the long-term effects of smoking and cessation on cancer risk.

Finally, this study may have been impacted by selection bias in the KNHANES data. Although the survey participation rate is relatively high, the sample may over-represent individuals who participate in National Health Insurance Service general health examinations, potentially leading to a biased estimation of smoking prevalence. Future studies could address this issue by incorporating alternative data sources, such as cigarette sales data, to improve the accuracy and generalizability of the prevalence estimates.

In conclusion, in 2015 tobacco smoking accounted for approximately one-quarter of cancer cases and one-third of cancer deaths in male. Although it was responsible for only about 1% of cancer cases and 5% of cancer deaths in female, the proportion of cancer caused by tobacco smoking in female is expected to increase by 2030.

## Figures and Tables

**Figure f5-epih-47-e2025008:**
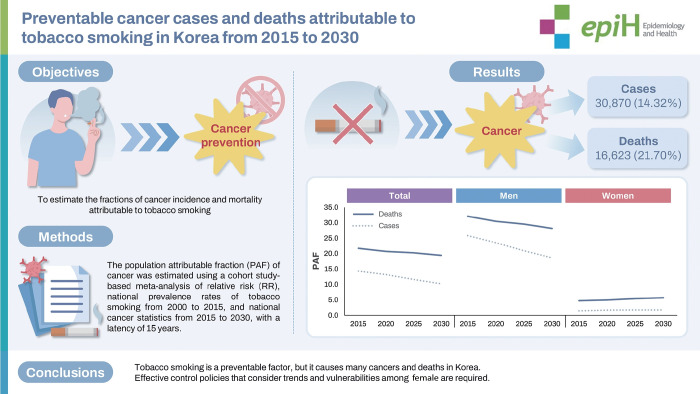


**Table 1.** PAF^1^ and cancer cases and deaths attributable to tobacco smoking in Korea in 2015 and 2020

Variables	Cancer incidence				Cancer mortality			
	2015		2020		2015		2020	
	PAF (%)	AC (n)	PAF (%)	AC (n)	PAF (%)	AC (n)	PAF (%)	AC (n)
Total population								
Lung	53.36	13,127	51.14	14,802	56.53	9,834	56.21	10,496
Larynx	55.88	645	53.86	648	59.73	205	57.59	184
Oral cavity/Pharynx	18.64	617	17.42	703	30.69	357	29.35	377
Esophagus	29.30	716	27.28	750	38.20	585	36.31	568
Stomach	24.75	7,275	23.63	6,300	16.16	1,377	15.16	1,138
Colorectum	11.19	3,035	10.61	3,745	8.03	666	7.70	683
Liver	17.44	2,772	16.00	2,421	18.94	2,142	17.53	1,852
Pancreas	16.27	1,038	15.22	1,277	16.13	878	15.03	1,019
Cervix uteri	2.54	92	2.99	90	6.47	63	7.43	60
Ovary	0.51	12	0.53	15	0.51	5	0.89	7
Kidney	4.00	225	3.55	262	13.58	182	11.70	194
Bladder	32.00	1,316	30.52	1,450	25.31	329	24.81	395
All cancer	14.32	30,870	13.17	32,463	21.70	16,623	20.69	16,973
Male								
Lung	72.27	12,447	70.55	13,865	71.92	9,117	70.18	9,701
Larynx	56.73	617	54.41	621	59.96	191	57.55	178
Oral cavity/Pharynx	24.50	588	23.04	663	38.41	338	36.39	354
Esophagus	30.47	678	28.40	696	37.62	527	35.10	493
Stomach	36.58	7,202	34.86	6,229	23.43	1,290	21.83	1,049
Colorectum	17.81	2,868	16.82	3,491	12.48	586	11.65	586
Liver	22.32	2,640	20.45	2,279	24.02	2,013	22.01	1,719
Pancreas	25.67	868	23.95	1,033	24.50	713	22.80	787
Kidney	5.41	208	4.69	239	18.34	169	15.63	175
Bladder	38.40	1,268	36.23	1,386	32.72	314	30.64	378
All cancer	25.83	29,384	23.49	30,502	32.09	15,258	30.41	15,420
Female								
Lung	9.22	680	10.08	937	15.19	717	16.41	795
Larynx	42.01	28	43.88	27	56.71	14	58.63	6
Oral cavity/Pharynx	3.21	29	3.47	40	6.84	19	7.31	23
Esophagus	17.41	38	18.03	54	44.48	58	46.99	75
Stomach	0.75	73	0.81	71	2.89	87	3.30	89
Colorectum	1.51	167	1.75	254	2.22	80	2.53	97
Liver	3.23	132	3.56	142	4.40	129	4.83	133
Pancreas	5.68	170	5.97	244	6.51	165	6.97	232
Cervix uteri	2.54	92	2.99	90	6.47	63	7.43	60
Ovary	0.51	12	0.53	15	0.51	5	0.89	7
Kidney	0.97	17	1.02	23	3.14	13	3.55	19
Bladder	5.89	48	6.94	64	4.34	15	4.73	17
All cancer	1.46	1,486	1.68	1,961	4.70	1,365	4.96	1,553

PAF, population attributable fraction; AC, attributable cancer cases or deaths.

1 The PAF was estimated using the number of cancers in the population in the given year with consistent relative risks and a 15-year latency period, along with the prevalence of tobacco smoking in 2000, 2005, 2010, and 2015, depending on the year of the estimate.

**Table 2.** Comparison of PAF (%) estimates using different RRs for cancers attributable to tobacco smoking

Variables	Cancer incidence					Cancer mortality				
	RR^1^			RR per mean Pys	RR for all cancers^1^	RR^1^			RR per mean Pys	RR for all cancers^1^
	Korean	Asian	Global			Korean	Asian	Global		
Total population										
Lung	53.36	53.01	63.69	27.19		56.53	53.03	57.57	26.17	
Larynx	55.88	60.78	60.78	25.06		59.73	59.73	63.92	42.18	
Oral cavity/Pharynx	18.64	21.31	30.43	13.86		30.69	34.24	42.91	16.96	
Esophagus	29.30	34.59	42.16	16.95		38.20	50.27	50.32	18.82	
Stomach	24.75	24.13	24.55	12.23		16.16	16.16	16.24	8.06	
Colorectum	11.19	11.19	10.17	5.33		8.03	7.68	10.46	3.76	
Liver	17.44	21.94	21.77	6.87		18.94	23.15	24.79	6.09	
Pancreas	16.27	16.53	22.24	7.12		16.13	18.59	19.19	9.34	
Cervix uteri	2.54	2.54	4.26	1.66		6.47	8.24	8.24	3.05	
Ovary	0.51	0.51	0.88	0.09		0.51	0.51	0.88	0.09	
Kidney	4.00	3.92	14.00	3.87		13.58	13.95	15.58	6.31	
Bladder	32.00	26.94	44.23	15.87		25.31	29.68	38.02	13.57	
All cancers	14.32	14.56	16.72	7.13	12.01	21.70	22.05	23.91	10.01	26.18
Male										
Lung	72.27	71.31	80.55	37.31		71.92	67.61	72.13	34.03	
Larynx	56.73	61.94	61.94	26.04		59.96	59.96	64.01	44.58	
Oral cavity/Pharynx	24.50	28.09	40.62	18.14		38.41	43.13	51.84	21.25	
Esophagus	30.47	36.28	45.12	18.35		37.62	52.52	52.52	19.12	
Stomach	36.58	35.65	36.05	18.08		23.43	23.43	23.43	11.66	
Colorectum	17.81	17.81	16.37	8.50		12.48	11.86	16.28	5.82	
Liver	22.32	28.34	28.34	8.79		24.02	29.53	32.06	7.47	
Pancreas	25.67	26.60	36.76	12.40		24.50	27.70	30.38	15.95	
Kidney	5.41	5.29	19.79	5.23		18.34	18.95	22.77	8.62	
Bladder	38.40	32.10	51.75	18.79		32.72	38.63	47.35	17.65	
All cancers	25.83	26.24	29.30	12.98	21.69	32.09	32.71	35.40	15.78	39.21
Female										
Lung	9.22	10.30	24.32	3.56		15.19	13.86	16.67	5.07	
Larynx	42.01	42.01	42.01	9.10		56.71	56.71	63.06	11.63	
Oral cavity/Pharynx	3.21	3.48	3.63	2.58		6.84	6.77	13.26	3.71	
Esophagus	17.41	17.41	12.10	2.64		44.48	26.02	26.02	15.52	
Stomach	0.75	0.75	1.19	0.35		2.89	2.89	2.89	1.50	
Colorectum	1.51	1.51	1.11	0.69		2.22	2.22	2.90	1.07	
Liver	3.23	3.30	3.26	1.30		4.40	4.90	4.59	2.14	
Pancreas	5.68	5.18	5.89	1.19		6.51	8.13	6.31	1.74	
Cervix uteri	2.54	2.54	4.26	1.66		6.47	8.24	8.24	3.05	
Ovary	0.51	0.51	0.88	0.09		0.51	0.51	0.88	0.09	
Kidney	0.97	0.97	1.54	0.95		3.14	2.98	2.19	1.25	
Bladder	5.89	5.89	43.97	3.97		4.34	4.34	9.58	2.02	
All cancers	1.46	1.52	2.67	0.60	1.20	4.70	4.62	5.12	1.74	4.88

PAF, population-attributable fraction; RR, relative risk; PYs, pack-years.

1 Tobacco smoking status was classified as current smoking, past smoking, or never smoking.

## Supplementary materials

Supplementary Material 1. Cancers caused by tobacco smoking

Supplementary Material 2. Prevalence rates^1^ of exposure to tobacco smoking in Korea

Supplementary Material 3. Cohort studies^1,2^ included in the meta-analysis for the association with tobacco smoking on the risk of specific cancer

Supplementary Material 4. Meta-analyzed relative risks and 95% confidence intervals for the risk of specific cancer according to tobacco smoking based on cohort studies in male

Supplementary Material 5. Meta-analyzed relative risks and 95% confidence intervals for the risk of specific cancer according to tobacco smoking based on cohort studies in female

Supplementary Material 6. Meta-analyzed relative risks and 95% CIs for the risk of specific cancer of tobacco smoking per 10 pack-years in Korean cohort studies

Supplementary Material 7. The population attributable fraction (%) of cancer cases attributed to tobacco smoking and proportion of specific cancers among all-cancer cases caused by tobacco smoking in Korea, 2020.

Supplementary Material 8. The population attributable fraction (%) of cancer deaths attributed to tobacco smoking and proportion of specific cancers among all-cancer deaths caused by tobacco smoking in Korea, 2020.

Supplementary Material 9. The population attributable fraction (%) of cancer cases attributed to tobacco smoking and proportion of specific cancers among all-cancer cases caused by tobacco smoking in Korea, 2015.

Supplementary Material 10. The population attributable fraction (%) of cancer deaths attributed to tobacco smoking and proportion of specific cancers among all-cancer deaths caused by tobacco smoking in Korea, 2015.

Supplementary Material 11. Comparison of population attributable fraction (PAF, %) in specific cancer attributed to tobacco smoking when using different relative risks (RRs).

Supplementary Material 12. Comparison between cancer PAFs1 caused by tobacco2 in 2009 and 2015
