# Supplementary material for: Preventable cancer cases and deaths attributable to tobacco smoking in Korea from 2015 to 2030
Source: Epidemiol Health. 2025 Feb 27;47:e2025008. doi: 10.4178/epih.e2025008 (PMC12531467; doi:10.4178/epih.e2025008)
Supplement: Supplementary Material 1. — Cancers caused by tobacco smoking [file epih-47-e2025008-Supplementary-1.docx]

Supplementary Material 1. Cancers caused by tobacco smoking

| **Risk factor** | **Cancers** | **Selection criteria** |
| --- | --- | --- |
| Tobacco smoking | Mouth, pharynx, and larynx (MPL) (C00-C14, C32)  Esophagus (C15)  Stomach (C16)  Colorectal (C18-C20)  Liver (C22)  Pancreas (C25)  Lung (C33-C34)  Cervix (C53)  Ovary (C56)  Kidney (C64-C66)  Bladder (C67) | IARC Group 1:  Carcinogenic to humans |

Hematopoietic cancer was deleted due to lack of Korean cohort studies.
