# Supplementary material for: Preventable cancer cases and deaths attributable to tobacco smoking in Korea from 2015 to 2030
Source: Epidemiol Health. 2025 Feb 27;47:e2025008. doi: 10.4178/epih.e2025008 (PMC12531467; doi:10.4178/epih.e2025008)
Supplement: Supplementary Material 2. — Prevalence rates1 of exposure to tobacco smoking in Korea [file epih-47-e2025008-Supplementary-2.docx]

Supplementary Material 2. Prevalence rates^1^ of exposure to tobacco smoking in Korea

|  | **Projected** | **Observed** | | | | **Projected** | |
| --- | --- | --- | --- | --- | --- | --- | --- |
|  | **2000** | **2005** | **2010** | **2015** | **2020** | **2025** | **2030** |
| **Current smoking** |  |  |  |  |  |  |  |
| Male | 62.3 | 55.3 | 48.3 | 39.9 | 32.9 | 25.9 | 18.9 |
| Female | 5.9 | 6.1 | 6.2 | 6.4 | 6.5 | 6.7 | 6.8 |
| **Past smoking** |  |  |  |  |  |  |  |
| Male | 21.1 | 25.2 | 29.2 | 30.6 | 34.6 | 38.7 | 42.7 |
| Female | 3.3 | 4.6 | 5.9 | 6.1 | 6.2 | 6.3 | 6.4 |

1. Prevalence rates of exposure to tobacco smoking (Standardized by 2000 mid-year Korean population) for projected years from 2015 to 2030 were estimated by linear regression model using Korea National Health and Nutrition Examination Survey data from 1998 to 2000
