# Supplementary material for: Preventable cancer cases and deaths attributable to tobacco smoking in Korea from 2015 to 2030
Source: Epidemiol Health. 2025 Feb 27;47:e2025008. doi: 10.4178/epih.e2025008 (PMC12531467; doi:10.4178/epih.e2025008)
Supplement: Supplementary Material 3. — Cohort studies1,2 included in the meta-analysis for the association with tobacco smoking on the risk of specific cancer [file epih-47-e2025008-Supplementary-3.docx]

Supplementary Material 3. Cohort studies^1,2^ included in the meta-analysis for the association with tobacco smoking on the risk of specific cancer

| **Oral cavity/pharynx** |  | **Larynx** |  |
| --- | --- | --- | --- |
| **Incidence** | **Death** | **Incidence** | **Death** |
| McLaughlin (1995) | Wen et al. (2004) | McLaughlin (1995) | Carter et al. (2015) |
|  | Carter et al. (2015) |  |  |
| Lu et al. (2018) | Liaw et al. (1998) | Lu et al. (2018) |  |
|  | Katanoda et al. (2008) |  |  |
| KMCC | KNHIS | KMCC | KNHIS |
| NWS/DGS | KSCS | KNHIS | KoGES |
| KNHIS | KoGES | KSCS | KMCC^3^ |
| KSCS | NWS/DGS^3^ | KNCC | NWS/DGS^3^ |
| KNCC |  | KSCP-II | KSCS^3^ |
| KSCP-II |  | NWS/DGS^3^ |  |
| H-PEACE^3^ |  | H-PEACE^3^ |  |
| **Esophageal cancer** |  | **Stomach cancer** |  |
| **Incidence** | **Death** | **Incidence** | **Death** |
| Zendehdel et al. (2008) |  | Freedman et al. (2007) |  |
| McLaughlin et al. (1995) |  | Nomura et al. (2012) |  |
| Nordlund et al. (1997) |  |  |  |
|  |  |  |  |
| Fan et al. (2008) | Zheng-Ming et al. (1997) | Galanis et al. (1998) |  |
| Ishiguro et al. (2009) | Katanoda et al. (2008) | Nomura et al. (1990) |  |
|  | Liaw et al. (1998) | Sasazuki et al. (2002) |  |
|  | Wen et al. (2004) | Jayalekshmi et al. (2015) |  |
|  | Yaegashi et al. (2014) | Moy et al. (2010) |  |
| KMCC | KMCC | KMCC | KMCC |
| NWS/DGS | KNHIS | NWS/DGS | KNHIS |
| KNHIS | KSCS | KNHIS | KSCS |
| KSCS | KCS | KSCS | KCS |
| KNCC | KoGES | KNCC | KoGES |
| KSCP-II |  | KSCP-II | KNHANES |
| H-PEACE |  | H-PEACE | SNUH-HPC |
| **Colorectal** |  | **Liver cancer** |  |
| **Incidence** | **Death** | **Incidence** | **Death** |
| Viner et al. (2019) | Carter et al. (2015) | Nordlund et al. (1997) | Carter et al. (2015) |
| Engeland et al. (1996) | Chao et al. (2000) | Petrick et al. (2018) |  |
| Bostick et al. (1994) | Heineman et al. (1994) |  |  |
| Nyrén et al. (1996) |  |  |  |
| Kato et al. (1997) |  |  |  |
| Nordlund et al. (1997) |  |  |  |
| Stürmer et al. (2000) |  |  |  |
| Poomphakwaen et al. | Liaw et al. (1998) | Goodman et al. (1995) | Zheng-Ming et al. (1997) |
| (2015) | Wen et al. (2004) | Koh et al. (2011) | Liaw et al. (1998) |
|  |  |  | Katanoda et al. (2008) |
|  |  |  | Mizoue et al. (2000) |
|  |  |  | Evans et al. (2002) |
|  |  |  |  |
|  |  |  |  |
| KMCC | KMCC | KMCC | KMCC |
| NWS/DGS | NWS/DGS | NWS/DGS | NWS/DGS |
| KNHIS | KNHIS | KNHIS | KNHIS |
| KSCS | KSCS | KSCS | KSCS |
| KNCC | KCS | KNCC | KCS |
| KSCP-II | KoGES | KSCP-II | KoGES |
| H-PEACE |  | H-PEACE | KNHANES |
|  |  |  | SNUH-HPC |
|  |  |  |  |
|  |  |  | (Continued) |
| **Pancreatic cancer** |  | **Lung cancer** |  |
| **Incidence** | **Death** | **Incidence** | **Death** |
| Arriaga et al. (2019) | Carter et al. (2015) | Hansen et al. (2017) | Carter et al. (2015) |
| Engeland et al. (1996) | Gapstur et al. (2000) | Viner et al. (2019) |  |
| Tulinius et al. (1997) | McLaughlin et al. | Nomura et al. (2018) |  |
| Harnack et al. (1997) | Nilsson et al. (2001) | Laaksonen et al. (2018) |  |
| Nilsen et al. (2000) | Tverdal et al. (1993) | Teleka et al. (2018) |  |
| Kuzmickiene et al. (2013) | Zheng et al. (1993) | McLaughlin et al. (1995) |  |
| Fuchs et al. (1996) |  | Tindle et al. (2018) |  |
| Nordlund et al. (1997) |  | Tulinius et al. (1997) |  |
| Stevens et al. (2009) |  | Engeland et al. (1996) |  |
|  |  | Nordlund et al. (1999) |  |
|  |  | Anderson et al. (1997) |  |
| Luo et al. (2007) | Katanoda et al. (2008) | Luo et al. (2007) | Zheng-Ming et al. (1997) |
| Pang et al. (2018) | Nakamura et al. (2010) | Zhang et al. (2016) | Ando et al. (2003) |
|  | Wen et al. (2004) |  | Chen et al. (2015) |
|  |  |  | Zha et al. (2019) |
|  |  |  | Wen et al. (2005) |
|  |  |  | Katanoda et al. (2008) |
|  |  |  | Tuvdendorj et al. (2020) |
| KMCC | KMCC | KMCC | KMCC |
| NWS/DGS | NWS/DGS | NWS/DGS | NWS/DGS |
| KNHIS | KNHIS | KNHIS | KNHIS |
| KSCS | KSCS | KSCS | KSCS |
| KNCC | KCS | KNCC | KCS |
| KSCP-II | KoGES | KSCP-II | KoGES |
| H-PEACE |  | H-PEACE | KNHANES |
| **Uterine cervix cancer** |  | **Ovarian cancer** |  |
| **Incidence** | **Death** | **Incidence** | **Death** |
| Engeland et al. (1996) |  | Licaj et al. (2017) | Carter et al. (2015) |
| Tulinius et al. (1997) |  | Nordlund et al. (1999) |  |
| Nordlund et al. (1999) |  | Tworoger et al. (2008) |  |
| Trimble et al. (2005) |  | Arthur et al. (2019) |  |
|  | Liaw et al. (1998) |  |  |
|  | Katanoda et al. (2008) |  |  |
|  | Wen et al. (2004) |  |  |
| KMCC | KMCC | KMCC | KMCC |
| NWS/DGS | KNHIS | KNHIS | KNHIS |
| KNHIS | KSCS | KSCS | KSCS |
| KSCS | KCS |  | KoGES |
| KNCC |  |  |  |
| KSCP-II |  |  |  |
| **Kidney cancer** |  | **Bladder cancer** |  |
| **Incidence** | **Death** | **Incidence** | **Death** |
| Engeland et al. (1996) | Carter et al. (2015) | Kurahashi et al. (2009) | Carter et al. (2015) |
| Flaherty et al. (2005) | Awamlh et al. (2019) | Engeland et al. (1996) |  |
| Setiawan et al. (2007) |  | Tulinius et al. (1997) |  |
| Nordlund et al. (1997) |  | Teleka et al. (2018) |  |
|  |  | Nordlund et al. (1997) |  |
|  |  | Freedman et al. (2011) |  |
|  |  | Bjerregaard et al. (2006) |  |
|  | Katanoda et al. (2008) |  | Liaw et al. (1998) |
|  | Wen et al. (2004) |  | Katanoda et al. (2008) |
|  |  |  | Wen et al. (2004) |
| KMCC | NWS/DGS | KMCC | KMCC |
| NWS/DGS | KNHIS | NWS/DGS | NWS/DGS |
| KNHIS | KSCS | KNHIS | KNHIS |
| KSCS | KoGES | KSCS | KSCS |
|  |  |  | (Continued) |
| KNCC | KoGES ^3^ | KNCC | KCS |
| KSCP-II |  | KSCP-II | KoGES^3^ |
| H-PEACE |  | H-PEACE |  |
| **All cancers ^2^** |  |  |  |
| **Incidence** | **Death** |  |  |
| KMCC | KMCC |  |  |
| NWS/DGS | NWS/DGS |  |  |
| KNHIS | KNHIS |  |  |
| KSCS | KSCS |  |  |
| KNCC | KCS |  |  |
| KSCP-II | KoGES |  |  |
| H-PEACE | KNHANES |  |  |
|  | SNUH-HPC |  |  |

Abbreviation: KNHIS-NHID, Korea National Health Insurance Service-National Health Information Database; KMCC, Korea Multicenter Cancer Cohort study; NWS/DGS, Namwon-Donggu study; KCPS-II, Korea Cancer Prevention Study-phase II; KNCC, Korea National Cancer Center study; H-PEACE, Health and Prevention Enhancement study; KSCS, Kangbuk Samsung Cohort Study. KCS, Kangwha Cohort Study; KoGES, Korea Genomic and Epidemiologic Study; KNHANES, Korea National Health And Nutritional Examination Survey-based Cohort; SNUH-HPC, Seoul National University Health Promotion Center Cohort. All cohort studies were described in prior paper (Lee S et al. J Prev Med Public Health 2022)

1. We wanted to calculate PAFs in 2015 and 2020 using the smoking prevalence rate in 2000 and 2005 (15-latency), respectively. Therefore, the literature selected in the systematic review was based on cohort studies from 1990 to 2019 that presented RR values for cancer risk attributed to tobacco smoking.

2. The RRs for all cancers and RRs per pack-year were calculated using raw-data analysis in Korean cohort studies.

3. Only used for pack-year analysis of tobacco smoking.

**[Reference for meta-analysis]**

1. Lu Y, Sobue T, Kitamura T, Matsuse R, Kitamura Y, Matsuo K, et al. Tobacco Smoking, alcohol drinking, and oral cavity and pharyngeal cancer in the Japanese: a population-based cohort study in Japan. European journal of cancer prevention: the official journal of the European Cancer Prevention Organisation (ECP). 2018;27(2):171-9.

2. McLaughlin JK, Hrubsec Z, Blot WJ, Fraumeni Jr JF. Smoking and cancer mortality among US veterans: a 26‐year follow‐up. International journal of cancer. 1995;60(2):190-3.

3. Nordlund LA, Carstensen JM, Pershagen G. Cancer incidence in female smokers: a 26‐year follow‐up. International journal of cancer. 1997;73(5):625-8.

4. Ishiguro S, Sasazuki S, Inoue M, Kurahashi N, Iwasaki M, Tsugane S, et al. Effect of alcohol consumption, Tobacco Smoking and flushing response on esophageal cancer risk: a population-based cohort study (JPHC study). Cancer letters. 2009;275(2):240-6.

5. Fan Y, Yuan J-M, Wang R, Gao Y-T, Yu MC. Alcohol, tobacco, and diet in relation to esophageal cancer: the Shanghai Cohort Study. Nutrition and cancer. 2008;60(3):354-63.

6. Zendehdel K, Nyrén O, Luo J, Dickman PW, Boffetta P, Englund A, et al. Risk of gastroesophageal cancer among smokers and users of Scandinavian moist snuff. International journal of cancer. 2008;122(5):1095-9.

7. Galanis DJ, Kolonel LN, Lee J, Nomura A. Intakes of selected foods and beverages and the incidence of gastric cancer among the Japanese residents of Hawaii: a prospective study. International Journal of Epidemiology. 1998;27(2):173-80.

8. Nomura A, Grove JS, Stemmermann GN, Severson RK. A prospective study of stomach cancer and its relation to diet, cigarettes, and alcohol consumption. Cancer Research. 1990;50(3):627-31.

9. Sasazuki S, Sasaki S, Tsugane S, Group JPHCS. Tobacco Smoking, alcohol consumption and subsequent gastric cancer risk by subsite and histologic type. International journal of cancer. 2002;101(6):560-6.

10. Jayalekshmi PA, Hassani S, Nandakumar A, Koriyama C, Sebastian P, Akiba S. Gastric cancer risk in relation to tobacco use and alcohol drinking in Kerala, India-Karunagappally cohort study. World journal of gastroenterology. 2015;21(44):12676.

11. Moy KA, Fan Y, Wang R, Gao Y-T, Mimi CY, Yuan J-M. Alcohol and tobacco use in relation to gastric cancer: a prospective study of men in Shanghai, China. Cancer Epidemiology and Prevention Biomarkers. 2010;19(9):2287-97.

12. Freedman ND, Abnet CC, Leitzmann MF, Mouw T, Subar AF, Hollenbeck AR, et al. A Prospective Study of Tobacco, Alcohol, and the Risk of Esophageal and Gastric Cancer Subtypes. American Journal of Epidemiology. 2007;165(12):1424-33.

13. Nomura AM, Wilkens LR, Henderson BE, Epplein M, Kolonel LN. The association of Tobacco Smoking with gastric cancer: the multiethnic cohort study. Cancer Causes & Control. 2012;23(1):51-8.

14.Viner B, Barberio AM, Haig TR, Friedenreich CM, Brenner DR. The individual and combined effects of alcohol consumption and Tobacco Smoking on site-specific cancer risk in a prospective cohort of 26,607 adults: results from Alberta’s Tomorrow Project. Cancer Causes & Control. 2019;30(12):1313-26.

15. Engeland A, Andersen A, Haldorsen T, Tretli S. Smoking habits and risk of cancers other than lung cancer: 28 years' follow-up of 26,000 Norwegian men and women. Cancer Causes & Control. 1996;7(5):497-506.

16. Bostick RM, Potter JD, Kushi LH, Sellers TA, Steinmetz KA, McKenzie DR, et al. Sugar, meat, and fat intake, and non-dietary risk factors for colon cancer incidence in Iowa women (United States). Cancer Causes & Control. 1994;5(1):38-52.

17. Nyrén O, Bergström R, Nyström L, Engholm G, Ekbom A, Adami H-O, et al. Smoking and Colorectal Cancer: a 20-Year Follow-up Study of Swedish Construction Workers. JNCI: Journal of the National Cancer Institute. 1996;88(18):1302-7.

18. Kato I, Akhmedkhanov A, Koenig K, Toniolo PG, Shore RE, Riboli E. Prospective study of diet and female colorectal cancer: the New York University Women's Health Study. 1997.

19. Stürmer T, Glynn RJ, Lee I-M, Christen WG, Hennekens CH. Lifetime Tobacco Smoking and colorectal cancer incidence in the Physicians' Health Study I. J Natl Cancer Inst. 2000;92(14):1178-81.

20. Jayalekshmi PA, Hassani S, Nandakumar A, Koriyama C, Sebastian P, Akiba S. Gastric cancer risk in relation to tobacco use and alcohol drinking in Kerala, India--Karunagappally cohort study. World J Gastroenterol. 2015 Nov 28;21(44):12676-85.

21. Poomphakwaen K, Promthet S, Suwanrungruang K, Kamsa-ard S, Wiangnon S. Risk Factors for Colorectal Cancer in Thailand. Asian Pac J Cancer Prev. 2015;16(14):6105-9.

22. Petrick JL, Campbell PT, Koshiol J, Thistle JE, Andreotti G, Beane-Freeman LE, et al. Tobacco, alcohol use and risk of hepatocellular carcinoma and intrahepatic cholangiocarcinoma: The Liver Cancer Pooling Project. Br J Cancer. 2018 Apr;118(7):1005-1012.

23. Koh WP, Robien K, Wang R, Govindarajan S, Yuan JM, Yu MC. Smoking as an independent risk factor for hepatocellular carcinoma: the Singapore Chinese Health Study. Br J Cancer. 2011 Oct 25;105(9):1430-5.

24. Goodman MT, Moriwaki H, Vaeth M, Akiba S, Hayabuchi H, Mabuchi K. Prospective cohort study of risk factors for primary liver cancer in Hiroshima and Nagasaki, Japan. Epidemiology. 1995:36-41.

25. Luo J, Iwasaki M, Inoue M, Sasazuki S, Otani T, Ye W, et al. Body mass index, physical activity and the risk of pancreatic cancer in relation to smoking status and history of diabetes: a large-scale population-based cohort study in Japan–the JPHC study. Cancer Causes & Control. 2007;18(6):603-12.

26. Arriaga ME, Vajdic CM, MacInnis RJ, Canfell K, Magliano DJ, Shaw JE, et al. The burden of pancreatic cancer in Australia attributable to smoking. Medical Journal of Australia. 2019;210(5):213

27. Tulinius H, Sigfússon N, Sigvaldason H, Bjarnadóttir K, Tryggvadóttir L. Risk factors for malignant diseases: a cohort study on a population of 22,946 Icelanders. Cancer epidemiology, biomarkers & prevention : a publication of the American Association for Cancer Research, cosponsored by the American Society of Preventive Oncology. 1997;6(11):863-73.

28. Harnack LJ, Anderson KE, Zheng W, Folsom AR, Sellers TA, Kushi LH. Smoking, alcohol, coffee, and tea intake and incidence of cancer of the exocrine pancreas: the Iowa Women's Health Study. Cancer Epidemiology and Prevention Biomarkers. 1997;6(12):1081-6.

29. Nilsen TIL, Vatten LJ. A prospective study of lifestyle factors and the risk of pancreatic cancer in Nord-Trøndelag, Norway. Cancer Causes & Control. 2000;11(7):645-52.

30. Kuzmickiene I, Everatt R, Virviciute D, Tamosiunas A, Radisauskas R, Reklaitiene R, et al. Smoking and other risk factors for pancreatic cancer: a cohort study in men in Lithuania. Cancer epidemiology. 2013;37(2):133-9.

31. Pang Y, Kartsonaki C, Turnbull I, Guo Y, Yang L, Bian Z, et al. Metabolic and lifestyle risk factors for acute pancreatitis in Chinese adults: a prospective cohort study of 0.5 million people. PLoS medicine. 2018;15(8):e1002618.

32. Fuchs CS, Colditz GA, Stampfer MJ, Giovannucci EL, Hunter DJ, Rimm EB, et al. A prospective study of Tobacco Smoking and the risk of pancreatic cancer. Archives of internal medicine. 1996;156(19):2255-60.

33. Stevens RJ, Roddam AW, Spencer EA, Pirie KL, Reeves GK, Green J, et al. Factors associated with incident and fatal pancreatic cancer in a cohort of middle-aged women. International journal of cancer. 2009;124(10):2400-5.

34. Zhang H, Ren J, Li N, Wang G, Guo L, Chen S, et al. [Prospective cohort study on the relationship between smoking cessation and cancer risk in males]. Zhonghua yu fang yi xue za zhi [Chinese journal of preventive medicine]. 2016;50(1):67-72.

35. Hansen MS, Licaj I, Braaten T, Langhammer A, Le Marchand L, Gram IT. Sex Differences in Risk of Smoking-Associated Lung Cancer: Results From a Cohort of 600,000 Norwegians. American Journal of Epidemiology. 2017;187(5):971-81.

36. Nomura SJO, Dash C, Rosenberg L, Palmer J, Adams-Campbell LL. Fruit and VegeTable Intake and Lung Cancer Incidence Among Black Women According to Tobacco Smoking Status. Nutrition and Cancer. 2018;70(6):904-12.

37. Laaksonen MA, Canfell K, MacInnis R, Arriaga ME, Banks E, Magliano DJ, et al. The future burden of lung cancer attributable to current modifiable behaviours: a pooled study of seven Australian cohorts. International Journal of Epidemiology. 2018;47(6):1772-83.

38. Tindle HA, Stevenson Duncan M, Greevy RA, Vasan RS, Kundu S, Massion PP, et al. Lifetime Smoking History and Risk of Lung Cancer: Results From the Framingham Heart Study. J Natl Cancer Inst. 2018;110(11):1201-7.

39. Teleka S, Häggström C, Nagel G, Bjørge T, Manjer J, Ulmer H, et al. Risk of bladder cancer by disease severity in relation to metabolic factors and smoking: A prospective pooled cohort study of 800,000 men and women. International journal of cancer. 2018;143(12):3071-82.

40. Nordlund LA, Carstensen JM, Pershagen G. Are male and female smokers at equal risk of smoking-related cancer: evidence from a Swedish prospective study. Scandinavian Journal of public health. 1999;27(1):56-62.

41. Anderson KE, Woo C, Olson JE, Sellers TA, Zheng W, Kushi LH, et al. Association of family history of cervical, ovarian, and uterine cancer with histological categories of lung cancer: the Iowa Women's Health Study. Cancer Epidemiology and Prevention Biomarkers. 1997;6(6):401-5.

42. Trimble CL, Genkinger JM, Burke AE, Hoffman SC, Helzlsouer KJ, Diener-West M, et al. Active and passive Tobacco Smoking and the risk of cervical neoplasia. Obstetrics and gynecology. 2005;105(1):174.

43. Licaj I, Jacobsen BK, Selmer RM, Maskarinec G, Weiderpass E, Gram IT. Smoking and risk of ovarian cancer by histological subtypes: an analysis among 300 000 Norwegian women. British journal of cancer. 2017;116(2):270-6.

44. Tworoger SS, Gertig DM, Gates MA, Hecht JL, Hankinson SE. Caffeine, alcohol, smoking, and the risk of incident epithelial ovarian cancer. Cancer: Interdisciplinary International Journal of the American Cancer Society. 2008;112(5):1169-77.

45. Arthur R, Brasky TM, Crane TE, Felix AS, Kaunitz AM, Shadyab AH, et al. Associations of a Healthy Lifestyle Index With the Risks of Endometrial and Ovarian Cancer Among Women in the Women's Health Initiative Study. Am J Epidemiol. 2019 Feb 1;188(2):261-273.

46. Flaherty KT, Fuchs CS, Colditz GA, Stampfer MJ, Speizer FE, Willett WC, et al. A prospective study of body mass index, hypertension, and smoking and the risk of renal cell carcinoma (United States). Cancer Causes & Control. 2005;16(9):1099-106.

47. Setiawan VW, Stram DO, Nomura AMY, Kolonel LN, Henderson BE. Risk Factors for Renal Cell Cancer: The Multiethnic Cohort. American Journal of Epidemiology. 2007;166(8):932-40.

48. Kurahashi N, Inoue M, Iwasaki M, Sasazuki S, Tsugane S, Group JPHCS. Coffee, green tea, and caffeine consumption and subsequent risk of bladder cancer in relation to smoking status: a prospective study in Japan. Cancer science. 2009;100(2):284-91.

49. Freedman ND, Silverman DT, Hollenbeck AR, Schatzkin A, Abnet CC. Association between smoking and risk of bladder cancer among men and women. Jama. 2011;306(7):737-45.

50. Al Hussein Al Awamlh B, Shoag JE, Ravikumar V, Posada L, Taylor BL, van der Mijn JC, et al. Association of Smoking and Death from Genitourinary Malignancies: Analysis of the National Longitudinal Mortality Study. J Urol. 2019 Dec;202(6):1248-1254.

51. Bjerregaard BK, Raaschou-Nielsen O, Sørensen M, Frederiksen K, Christensen J, Tjønneland A, et al. Tobacco smoke and bladder cancer--in the European Prospective Investigation into Cancer and Nutrition. Int J Cancer. 2006 Nov 15;119(10):2412-6.

51. Liaw KM, Chen CJ. Mortality attributable to Tobacco Smoking in Taiwan: a 12-year follow-up study. Tobacco control. 1998;7(2):141-8.

52. Katanoda K, Marugame T, Saika K, Satoh H, Tajima K, Suzuki T, et al. Population attributable fraction of mortality associated with tobacco smoking in Japan: a pooled analysis of three large-scale cohort studies. J Epidemiol. 2008;18(6):251–264.

53. Wen CP, Tsai SP, Chen CJ, Cheng TY. The mortality risks of smokers in Taiwan: Part I: cause-specific mortality. Preventive medicine. 2004;39(3):528-35.

54. Carter BD, Abnet CC, Feskanich D, Freedman ND, Hartge P, Lewis CE, et al. Smoking and mortality—beyond established causes. New England journal of medicine. 2015;372(7):631-40.

55. Zheng-Ming C, Xu Z, Collins R, Li W-X, Peto R. Early health effects of the emerging tobacco epidemic in China: a 16-year prospective study. Jama. 1997;278(18):1500-4.

56. Yaegashi Y, Onoda T, Morioka S, Hashimoto T, Takeshita T, Sakata K, et al. Joint effects of smoking and alcohol drinking on esophageal cancer mortality in Japanese men: findings from the Japan collaborative cohort study. Asian Pac J Cancer Prev. 2014;15(2):1023-9.

57. Chao A, Thun MJ, Jacobs EJ, Henley SJ, Rodriguez C, Calle EE. Tobacco Smoking and colorectal cancer mortality in the cancer prevention study II. J Natl Cancer Inst. 2000;92(23):1888-96.

58. Heineman EF, Zahm SH, McLaughlin JK, Vaught JB. Increased risk of colorectal cancer among smokers: results of a 26‐year follow‐up of US veterans and a review. International journal of cancer. 1994;59(6):728-38.

59. Mizoue T, Tokui N, Nishisaka K, Nishisaka S-i, Ogimoto I, Ikeda M, et al. Prospective study on the relation of Tobacco Smoking with cancer of the liver and stomach in an endemic region. International journal of epidemiology. 2000;29(2):232-7.

60. Evans AA, Chen G, Ross EA, Shen F-M, Lin W-Y, London WT. Eight-year follow-up of the 90,000-person Haimen City cohort: I. Hepatocellular carcinoma mortality, risk factors, and sex differences. Cancer Epidemiology and Prevention Biomarkers. 2002;11(4):369-76.

61. Nakamura K, Nagata C, Wada K, Tamai Y, Tsuji M, Takatsuka N, et al. Tobacco Smoking and Other Lifestyle Factors in Relation to the Risk of Pancreatic Cancer Death: A Prospective Cohort Study in Japan. Japanese Journal of Clinical Oncology. 2010;41(2):225-31.

62. Gapstur SM, Gann PH, Lowe W, Liu K, Colangelo L, Dyer A. Abnormal Glucose Metabolism and Pancreatic Cancer Mortality. JAMA. 2000;283(19):2552-8.

63. Nilsson S, Carstensen J, Pershagen G. Mortality among male and female smokers in Sweden: a 33 year follow up. Journal of Epidemiology & Community Health. 2001;55(11):825-30.

64. Tverdal A, Thelle D, Stensvold I, Leren P, Bjartveit K. Mortality in relation to smoking history: 13 years' follow-up of 68,000 Norwegian men and women 35–49 years. Journal of clinical epidemiology. 1993;46(5):475-87.

65. Zheng W, McLaughlin JK, Gridley G, Bjelke E, Schuman LM, Silverman DT, et al. A cohort study of smoking, alcohol consumption, and dietary factors for pancreatic cancer (United States). Cancer Causes & Control. 1993;4(5):477-82.

66. Ando M, Wakai K, Seki N, Tamakoshi A, Suzuki K, Ito Y, et al. Attributable and absolute risk of lung cancer death by smoking status: findings from the Japan Collaborative Cohort Study. International journal of cancer. 2003;105(2):249-54.

67. Chen ZM, Peto R, Iona A, Guo Y, Chen YP, Bian Z, et al. Emerging tobacco‐related cancer risks in China: A nationwide, prospective study of 0.5 million adults. Cancer. 2015;121(S17):3097-106.

68. Tuvdendorj A, Feenstra T, Tseveen B, Buskens E. Smoking-attributable burden of lung cancer in Mongolia a data synthesis study on differences between men and women. PloS one. 2020;15(2):e0229090.

69. Zha L, Sobue T, Kitamura T, Kitamura Y, Sawada N, Iwasaki M, et al. Changes in smoking status and mortality from all causes and lung cancer: A longitudinal analysis of a population-based study in Japan. J Epidemiol. 2019;29(1):11-7.

70. Wen C, Tsai S, Chen C, Cheng T, Tsai M, Levy D. Smoking attributable mortality for Taiwan and its projection to 2020 under different smoking scenarios. Tobacco control. 2005;14(suppl 1):i76-i80.
