# Supplementary material for: Preventable cancer cases and deaths attributable to tobacco smoking in Korea from 2015 to 2030
Source: Epidemiol Health. 2025 Feb 27;47:e2025008. doi: 10.4178/epih.e2025008 (PMC12531467; doi:10.4178/epih.e2025008)
Supplement: Supplementary Material 4. — Meta-analyzed relative risks and 95% confidence intervals for the risk of specific cancer according to tobacco smoking based on cohort studies in male [file epih-47-e2025008-Supplementary-4.docx]

Supplementary Material 4. Meta-analyzed relative risks and 95% confidence intervals for the risk of specific cancer according to tobacco smoking based on cohort studies in male

|  | **Tobacco** | **Male** | | | | | |
| --- | --- | --- | --- | --- | --- | --- | --- |
| **Cancers** | **smoking** | **Cancer incidence** | | | **Cancer death** | | |
|  |  | **Korean RR (95% CI)^1^** | **Asian RR (95% CI)^2^** | **Global RR (95% CI)^2^** | **Korean RR (95% CI)^1^** | **Asian RR (95% CI)^2^** | **Global RR (95% CI)^2^** |
| Oral cavity/ | Past | 1.17 (0.99-1.38) | 1.18 (1.02-1.37) | 1.23 (1.05-1.43) | 1.41 (1.13-1.75) | 1.37 (1.01-1.87) | 1.44 (1.12-1.86) |
| Pharynx | Current | 1.46 (1.10-1.94) | 1.57 (1.21-2.03) | 2.02 (1.52-2.69) | 1.90 (1.73-2.09) | 2.09 (1.66-2.63) | 2.58 (1.84-3.62) |
| Esophagus | Past | 1.12 (1.04-1.20) | 1.22 (0.82-1.83) | 1.39 (1.01-1.91) | 1.08 (1.00-1.19) | 1.75 (1.07-2.86) | 1.75 (1.07-2.86) |
|  | Current | 1.66 (1.01-2.73) | 1.84 (1.28-2.64) | 2.19 (1.53-3.12) | 1.94 (1.81-2.08) | 2.52 (1.84-3.46) | 2.52 (1.84-3.46) |
| Stomach | Past | 1.36 (1.18-1.57) | 1.43 (1.24-1.65) | 1.46 (1.28-1.67) | 1.12 (1.07-1.16) | 1.12 (1.07-1.16) | 1.12 (1.07-1.16) |
|  | Current | 1.80 (1.44-2.26) | 1.74 (1.47-2.08) | 1.75 (1.48-2.06) | 1.45 (1.41-1.50) | 1.45 (1.41-1.50) | 1.45 (1.41-1.50) |
| Colorectal | Past | 1.15 (1.03-1.29) | 1.15 (1.03-1.29) | 1.16 (1.06-1.28) | 1.08 (1.03-1.13) | 1.08 (1.03-1.13) | 1.20 (1.08-1.33) |
|  | Current | 1.30 (1.08-1.55) | 1.30 (1.08-1.55) | 1.26 (1.12-1.41) | 1.20 (1.16-1.25) | 1.19 (1.10-1.28) | 1.25 (1.17-1.32) |
| Liver | Past | 1.03 (1.01-1.06) | 1.21 (0.92-1.59) | 1.21 (0.92-1.59) | 1.02 (0.99-1.06) | 1.22 (0.95-1.55) | 1.27 (1.01-1.59) |
|  | Current | 1.45 (1.32-1.59) | 1.56 (1.31-1.87) | 1.56 (1.31-1.87) | 1.50 (1.43-1.57) | 1.60 (1.33-1.93) | 1.67 (1.39-2.00) |
| Pancreas | Past | 1.13 (1.07-1.19) | 1.13 (1.07-1.19) | 1.13 (1.07-1.19) | 1.11 (1.05-1.18) | 1.12 (1.06-1.18) | 1.11 (1.02-1.22) |
|  | Current | 1.51 (1.15-1.99) | 1.54 (1.39-1.70) | 1.89 (1.56-2.29) | 1.48 (1.20-1.83) | 1.58 (1.51-1.64) | 1.66 (1.50-1.84) |
| Larynx | Past | 1.45 (1.31-1.62) | 1.45 (1.31-1.62) | 1.45 (1.31-1.62) | 1.41 (1.13-1.75) | 1.41 (1.13-1.75) | 1.53 (1.27-1.83) |
|  | Current | 2.95 (2.73-3.19) | 2.95 (2.73-3.19) | 2.95 (2.73-3.19) | 3.27 (2.80-3.81) | 3.27 (2.80-3.81) | 3.68 (3.17-4.26) |
| Lung | Past | 2.28 (1.67-3.12) | 2.13 (1.62-2.79) | 2.58 (1.98-3.38) | 2.24 (1.66-3.01) | 2.17 (1.81-2.60) | 2.17 (1.81-2.60) |
|  | Current | 4.75 (3.39-6.66) | 4.61 (3.24-6.54) | 7.11 (4.57-11.07) | 4.69 (3.38-6.51) | 3.96 (3.42-4.58) | 4.76 (3.27-6.91) |
| Cervix uteri | Past |  |  |  |  |  |  |
|  | Current |  |  |  |  |  |  |
| Ovary | Past |  |  |  |  |  |  |
|  | Current |  |  |  |  |  |  |
| Kidney | Past | 0.99 (0.94-1.05) | 0.99 (0.94-1.05) | 1.11 (0.94-1.31) | 0.94 (0.58-1.54) | 1.03 (0.70-1.51) | 1.19 (0.81-1.76) |
|  | Current | 1.09 (1.05-1.14) | 1.09 (1.05-1.14) | 1.36 (1.08-1.17) | 1.36 (1.24-1.50) | 1.37 (1.24-1.50) | 1.41 (1.29-1.54) |
| Bladder | Past | 1.23 (1.17-1.29) | 1.23 (1.17-1.29) | 1.66 (1.32-2.09) | 1.16 (1.04-11.29) | 1.17 (1.05-1.30) | 1.58 (0.98-2.56) |
|  | Current | 1.92 (1.50-2.47) | 1.68 (1.49-1.89) | 2.50 (1.81-3.45) | 1.73 (1.59-1.88) | 1.70 (0.98-2.97) | 2.25 (1.36-3.71) |

Abbreviation: RR, Relative risk; CI, Confidence interval; I, RR was used for the RR in incidence data.

1. The Korean RRs were estimated by meta-analysis using RRs calculated by raw data analysis from Korean cohort studies participated in the Korean Cohort Consortium (Lee S et al., J Prev Med Pub Health, 2022).

2. Asian and Global RRs were estimated by meta-analysis using RRs extracted in systematic review for cohort study. Those studies were described in Appendix Table 3.
