# Supplementary material for: Preventable cancer cases and deaths attributable to tobacco smoking in Korea from 2015 to 2030
Source: Epidemiol Health. 2025 Feb 27;47:e2025008. doi: 10.4178/epih.e2025008 (PMC12531467; doi:10.4178/epih.e2025008)
Supplement: Supplementary Material 5. — Meta-analyzed relative risks and 95% confidence intervals for the risk of specific cancer according to tobacco smoking based on cohort studies in female [file epih-47-e2025008-Supplementary-5.docx]

Supplementary Material 5. Meta-analyzed relative risks and 95% confidence intervals for the risk of specific cancer according to tobacco smoking based on cohort studies in female

|  | **Tobacco** | **Female** | | | | | |
| --- | --- | --- | --- | --- | --- | --- | --- |
| **Cancers** | **smoking** | **Cancer incidence** | | | **Cancer death** | | |
|  |  | **Korean RR (95% CI)^1^** | **Asian RR (95% CI)^2^** | **Global RR (95% CI)^2^** | **Korean RRs^1^** | **Asian RRs^2^** | **Global RRs^2^** |
| Oral cavity/ | Past | 1.14 (0.74-1.76) | 1.14 (0.74-1.76) | 1.14 (0.74-1.76) | 1.25 (0.56-2.82) | 1.25 (0.59-2.65) | 2.01 (1.48-2.72) |
| Pharynx | Current | 1.49 (1.19-1.86) | 1.54 (1.23-1.92) | 1.56 (1.27-1.92) | 2.10 (1.49-2.97) | 2.09 (1.51-2.90) | 3.03 (2.34-3.92) |
| Esophagus | Past | 1.16 (0.55-2.46) | 1.16 (0.55-2.46) | 1.69 (0.60-4.78) | 5.90 (0.28-124.82) | 3.27 (1.09-9.80) | 3.27 (1.09-9.80) |
|  | Current | 4.48 (1.07-18.77) | 4.48 (1.07-18.77) | 2.95 (1.59-5.47) | 11.84 (2.42-57.98) | 5.69 (2.34-13.82) | 5.69 (2.34-13.82) |
| Stomach | Past | 1.03 (0.94-1.14) | 1.03 (0.94-1.14) | 1.02 (0.93-1.12) | 1.28 (1.07-1.53) | 1.28 (1.07-1.53) | 1.28 (1.07-1.53) |
|  | Current | 1.11 (0.78-1.59) | 1.11 (0.78-1.59) | 1.23 (0.90-1.67) | 1.35 (1.21-1.50) | 1.35 (1.21-1.50) | 1.35 (1.21-1.50) |
| Colorectal | Past | 1.16 (1.05-1.27) | 1.16 (1.05-1.27) | 1.15 (1.06-1.25) | 1.21 (1.01-1.45) | 1.21 (1.01-1.45) | 1.20 (1.09-1.33) |
|  | Current | 1.17 (0.97-1.42) | 1.17 (0.97-1.42) | 1.11 (0.99-1.24) | 1.27 (1.14-1.41) | 1.27 (1.14-1.41) | 1.39 (1.26-1.54) |
| Liver | Past | 1.21 (1.04-1.41) | 1.23 (1.06-1.42) | 1.23 (1.06-1.42) | 1.26 (1.05-1.53) | 1.41 (1.11-1.78) | 1.28 (1.07-1.53) |
|  | Current | 1.45 (1.32-1.58) | 1.45 (1.33-1.59) | 1.44 (1.32-1.58) | 1.63 (1.47-1.82) | 1.65 (1.49-1.82) | 1.66 (1.51-1.83) |
| Pancreas | Past | 1.10 (0.91-1.33) | 1.11 (0.92-1.34) | 1.17 (1.02-1.33) | 1.24 (1.02-1.51) | 1.32 (1.10-1.58) | 1.24 (1.12-1.37) |
|  | Current | 1.96 (1.23-3.14) | 1.86 (1.30-2.67) | 1.97 (1.60-2.40) | 2.04 (1.27-3.28) | 2.32 (1.55-3.47) | 2.01 (1.62-2.48) |
| Larynx | Past | 3.77 (1.62-8.76) | 3.77 (1.62-8.76) | 3.77 (1.62-8.76) | 6.26 (1.47-26.65) | 6.26 (1.47-26.65) | 8.51 (3.05-23.765) |
|  | Current | 11.73 (8.28-16.60) | 11.73 (8.28-16.60) | 11.73 (8.28-16.60) | 20.26 (11.08-37.03) | 20.26 (11.08-37.03) | 25.74 (14.74-44.93) |
| Lung | Past | 1.60 (1.24-2.07) | 1.54 (1.20-1.99) | 2.23 (1.76-2.83) | 1.93 (1.33-2.79) | 20.26 (11.08-37.03) | 2.06 (1.80-2.36) |
|  | Current | 2.39 (1.80-3.16) | 2.64 (1.91-3.65) | 5.76 (3.40-9.75) | 3.52 (3.22-3.84) | 3.13 (2.66-3.69) | 3.80 (2.02-7.15) |
| Cervix uteri | Past | 1.33 (1.14-1.55) | 1.33 (1.14-1.55) | 1.29 (1.12-1.50) | 1.74 (0.79-3.84) | 1.43 (0.92-2.24) | 1.43 (0.92-2.24) |
|  | Current | 1.26 (1.12-1.41) | 1.26 (1.12-1.41) | 1.59 (1.12-2.25) | 1.76 (1.04-2.97) | 2.28 (1.43-3.63) | 2.28 (1.43-3.63) |
| Ovary | Past | 0.97 (0.77-1.21) | 0.97 (0.77-1.21) | 1.12 (0.98-1.26) | 0.97 (0.77-1.21) ^I^ | 0.97 (0.77-1.21) ^I^ | 1.12 (0.98-1.26) ^I^ |
|  | Current | 1.09 (0.94-1.26) | 1.09 (0.94-1.26) | 1.15 (0.92-1.42) | 1.09 (0.94-1.26) ^I^ | 1.09 (0.94-1.26) ^I^ | 1.15 (0.92-1.42) ^I^ |
| Kidney | Past | 1.02 (0.77-1.35) | 1.02 (0.77-1.35) | 1.19 (0.99-1.44) | 1.28 (0.66-2.48) | 1.30 (0.69-2.45) | 1.21 (1.08-1.42) |
|  | Current | 1.16 (0.95-1.40) | 1.16 (0.95-1.40) | 1.16 (0.99-1.35) | 1.39 (0.94-2.07) | 1.35 (0.91-1.99) | 1.26 (0.98-1.64) |
| Bladder | Past | 1.84 (0.93-3.63) | 1.84 (0.93-3.63) | 2.06 (1.57-2.71) | 1.23 (0.63-2.38) | 1.23 (0.63-2.38) | 1.82 (1.00-3.30) |
|  | Current | 1.59 (1.32-1.92) | 1.59 (1.32-1.92) | 3.06 (2.07-4.51) | 1.64 (1.17-2.30) | 1.64 (1.17-2.30) | 2.34 (1.22-4.48) |

Abbreviation: RR, Relative risk; CI, Confidence interval; I, RR was used for the RR in incidence data.

1. The Korean RRs were estimated by meta-analysis using RRs calculated by raw data analysis from Korean cohort studies participated in the Korean Cohort Consortium (Lee S et al., J Prev Med Pub Health, 2022).

2. Asian and Global RRs were estimated by meta-analysis using RRs extracted in systematic review for cohort study. Those studies were described in Appendix Table 3.
