# Supplementary material for: Preventable cancer cases and deaths attributable to tobacco smoking in Korea from 2015 to 2030
Source: Epidemiol Health. 2025 Feb 27;47:e2025008. doi: 10.4178/epih.e2025008 (PMC12531467; doi:10.4178/epih.e2025008)
Supplement: Supplementary Material 6. — Meta-analyzed relative risks and 95% CIs for the risk of specific cancer of tobacco smoking per 10 pack-years in Korean cohort studies [file epih-47-e2025008-Supplementary-6.docx]

Supplementary Material 6. Meta-analyzed relative risks and 95% CIs for the risk of specific cancer of tobacco smoking per 10 pack-years in Korean cohort studies

|  | **Male** | | **Female** | |
| --- | --- | --- | --- | --- |
| **Cancers** | **Cancer incidence** | **Cancer death** | **Cancer incidence** | **Cancer death** |
|  | **RRs per 10 Pack-years** | | **RRs per 10 Pack-years** | |
| Oral cavity/ Pharynx | 1.15 (1.10-1.20) | 1.17 (1.13-1.22) | 1.21 (1.05-1.38) | 1.33 (1.10-1.60) |
| Esophagus | 1.17 (1.08-1.27) | 1.18 (1.16-1.21) | 1.55 (1.38-1.73) | 1.56 (1.34-1.80) |
| Stomach | 1.13 (1.09-1.16) | 1.09 (1.08-1.10) | 1.08 (1.04-1.13) | 1.14 (1.07-1.21) |
| Colorectal | 1.05 (1.02-1.09) | 1.04 (1.03-1.05) | 1.04 (1.00-1.09) | 1.09 (1.02-1.17) |
| Liver | 1.08 (1.08-1.09) | 1.07 (1.04-1.10) | 1.15 (1.09-1.21) | 1.32 (1.06-1.65) |
| Pancreas | 1.10 (1.03-1.18) | 1.14 (1.12-1.15) | 1.21 (1.14-1.28) | 1.23 (1.17-1.30) |
| Larynx | 1.27 (1.20-1.36) | 1.32 (1.28-1.37) | 1.93 (1.72-2.14) | 2.06 (1.77-2.39) |
| Lung | 1.27 (1.20-1.34) | 1.21 (1.14-1.29) | 1.43 (1.31-1.55) | 1.58 (1.54-1.61) |
| Cervix uteri |  |  | 1.15 (1.06-1.24) | 2.06 (1.77-2.39) |
| Ovary |  |  | 1.00 (0.99-1.01) | 1.00 (0.99-1.01) ^I^ |
| Kidney | 1.05 (1.00-1.10) | 1.08 (1.05-1.12) | 1.22 (1.00-1.48) | 1.08 (0.83-1.41) |
| Bladder | 1.16 (1.14-1.17) | 1.16 (1.13-1.18) | 1.34 (1.23-1.45) | 1.32 (1.14-1.54) |
|  |  |  |  |  |
|  | **RR for [Current, Past smoking] vs. [Never smoking] on the risk of all cancers** | | | |
| All cancer |  |  |  |  |
| Past smoking | 1.12 (1.00-1.24) | 1.32 (1.19-1.47) | 1.07 (0.92-1.23) | 1.34 (1.05-1.70) |
| Current smoking | 1.41 (1.26-1.57) | 1.98 (1.73-2.15) | 1.17 (1.10-1.25) | 1.68 (1.50-1.88) |

Abbreviation: RR, Relative risk; CI, Confidence interval; I, RR was used for the RR in incidence data.

1. The Korean RRs were meta-analyzed using individual RRs calculated by data analysis from the Korean cohort studies participated in the Korean Cohort Consortium. For cancer death, 8 cohort studies were included as follows: KNHIS; KMCC; NWS/DGS; KSCS; Kangwha Cohort Study (KCS); Korea Genomic and Epidemiologic Study (KoGES); Korea National Health And Nutritional Examination Survey-based Cohort (KNHANES); Seoul National University Health Promotion Center Cohort (SNUH-HPC).
