# Supplementary material for: Preventable cancer cases and deaths attributable to tobacco smoking in Korea from 2015 to 2030
Source: Epidemiol Health. 2025 Feb 27;47:e2025008. doi: 10.4178/epih.e2025008 (PMC12531467; doi:10.4178/epih.e2025008)
Supplement: Supplementary Material 7. — The population attributable fraction (%) of cancer cases attributed to tobacco smoking and proportion of specific cancers among all-cancer cases caused by tobacco smoking in Korea, 2020. [file epih-47-e2025008-Supplementary-7.pptx]

## Slide 1
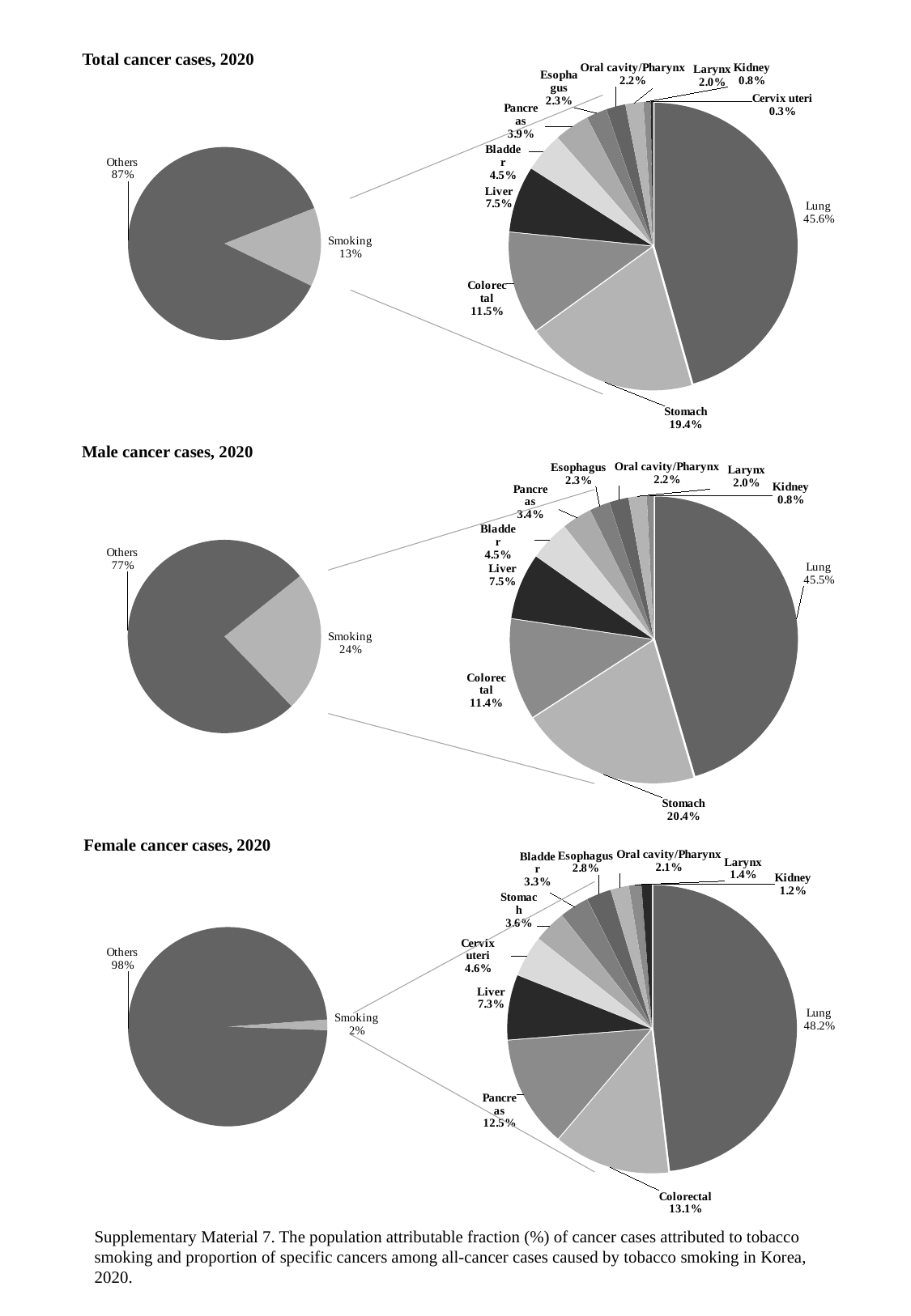

Total cancer cases, 2020
### Chart
| Category | |
|---|---|
| Lung | 14802.0 |
| Stomach | 6300.0 |
| Colorectal | 3745.0 |
| Liver | 2421.0 |
| Bladder | 1450.0 |
| Pancreas | 1277.0 |
| Esophagus | 750.0 |
| Oral cavity/Pharynx | 703.0 |
| Larynx | 648.0 |
| Kidney | 262.0 |
| Cervix uteri | 90.0 |
### Chart
| Category | |
|---|---|
| Others | 86.8 |
| Smoking | 13.2 |Male cancer cases, 2020
### Chart
| Category | |
|---|---|
| Lung | 13865.0 |
| Stomach | 6229.0 |
| Colorectal | 3491.0 |
| Liver | 2279.0 |
| Bladder | 1386.0 |
| Pancreas | 1033.0 |
| Esophagus | 696.0 |
| Oral cavity/Pharynx | 663.0 |
| Larynx | 621.0 |
| Kidney | 239.0 |
### Chart
| Category | |
|---|---|
| Others | 76.5 |
| Smoking | 23.5 |Female cancer cases, 2020
### Chart
| Category | |
|---|---|
| Lung | 937.0 |
| Colorectal | 254.0 |
| Pancreas | 244.0 |
| Liver | 142.0 |
| Cervix uteri | 90.0 |
| Stomach | 71.0 |
| Bladder | 64.0 |
| Esophagus | 54.0 |
| Oral cavity/Pharynx | 40.0 |
| Larynx | 27.0 |
| Kidney | 23.0 |
### Chart
| Category | |
|---|---|
| Others | 98.3 |
| Smoking | 1.7 |Supplementary Material 7. The population attributable fraction (%) of cancer cases attributed to tobacco smoking and proportion of specific cancers among all-cancer cases caused by tobacco smoking in Korea, 2020.
