# Supplementary material for: Preventable cancer cases and deaths attributable to tobacco smoking in Korea from 2015 to 2030
Source: Epidemiol Health. 2025 Feb 27;47:e2025008. doi: 10.4178/epih.e2025008 (PMC12531467; doi:10.4178/epih.e2025008)
Supplement: Supplementary Material 8. — The population attributable fraction (%) of cancer deaths attributed to tobacco smoking and proportion of specific cancers among all-cancer deaths caused by tobacco smoking in Korea, 2020. [file epih-47-e2025008-Supplementary-8.pptx]

## Slide 1
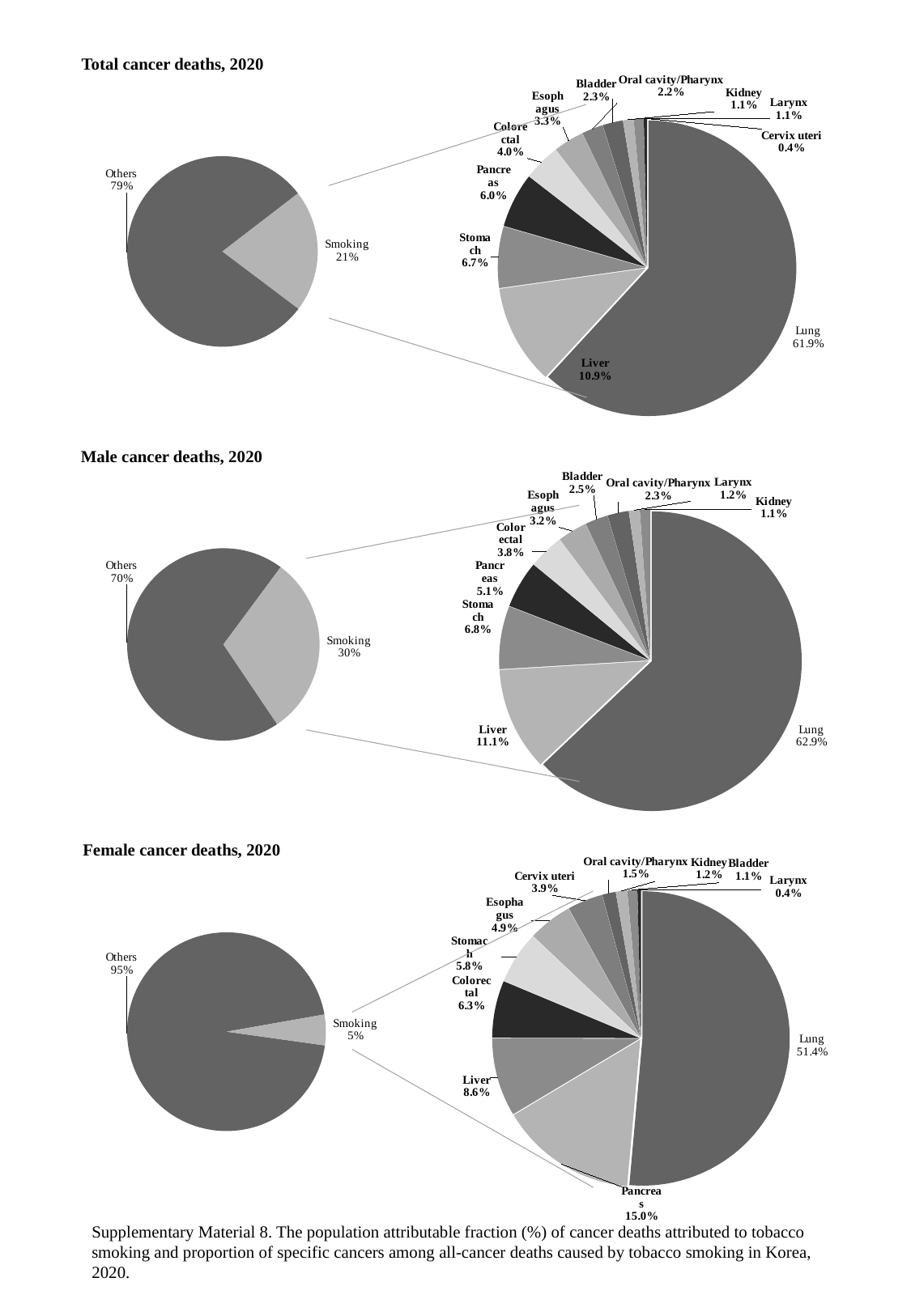

Total cancer deaths, 2020
### Chart
| Category | |
|---|---|
| Lung | 10497.0 |
| Liver | 1852.0 |
| Stomach | 1139.0 |
| Pancreas | 1018.0 |
| Colorectal | 683.0 |
| Esophagus | 568.0 |
| Bladder | 395.0 |
| Oral cavity/Pharynx | 377.0 |
| Kidney | 194.0 |
| Larynx | 185.0 |
| Cervix uteri | 60.0 |
### Chart
| Category | |
|---|---|
| Others | 79.3 |
| Smoking | 20.7 |Male cancer deaths, 2020
### Chart
| Category | |
|---|---|
| Lung | 9701.0 |
| Liver | 1719.0 |
| Stomach | 1049.0 |
| Pancreas | 787.0 |
| Colorectal | 586.0 |
| Esophagus | 493.0 |
| Bladder | 378.0 |
| Oral cavity/Pharynx | 354.0 |
| Larynx | 178.0 |
| Kidney | 175.0 |
### Chart
| Category | |
|---|---|
| Others | 69.6 |
| Smoking | 30.4 |Female cancer deaths, 2020
### Chart
| Category | |
|---|---|
| Lung | 795.0 |
| Pancreas | 232.0 |
| Liver | 133.0 |
| Colorectal | 97.0 |
| Stomach | 89.0 |
| Esophagus | 75.0 |
| Cervix uteri | 60.0 |
| Oral cavity/Pharynx | 23.0 |
| Kidney | 19.0 |
| Bladder | 17.0 |
| Larynx | 6.0 |
### Chart
| Category | |
|---|---|
| Others | 95.0 |
| Smoking | 5.0 |Supplementary Material 8. The population attributable fraction (%) of cancer deaths attributed to tobacco smoking and proportion of specific cancers among all-cancer deaths caused by tobacco smoking in Korea, 2020.
