# Supplementary material for: Preventable cancer cases and deaths attributable to tobacco smoking in Korea from 2015 to 2030
Source: Epidemiol Health. 2025 Feb 27;47:e2025008. doi: 10.4178/epih.e2025008 (PMC12531467; doi:10.4178/epih.e2025008)
Supplement: Supplementary Material 9. — The population attributable fraction (%) of cancer cases attributed to tobacco smoking and proportion of specific cancers among all-cancer cases caused by tobacco smoking in Korea, 2015. [file epih-47-e2025008-Supplementary-9.pptx]

## Slide 1
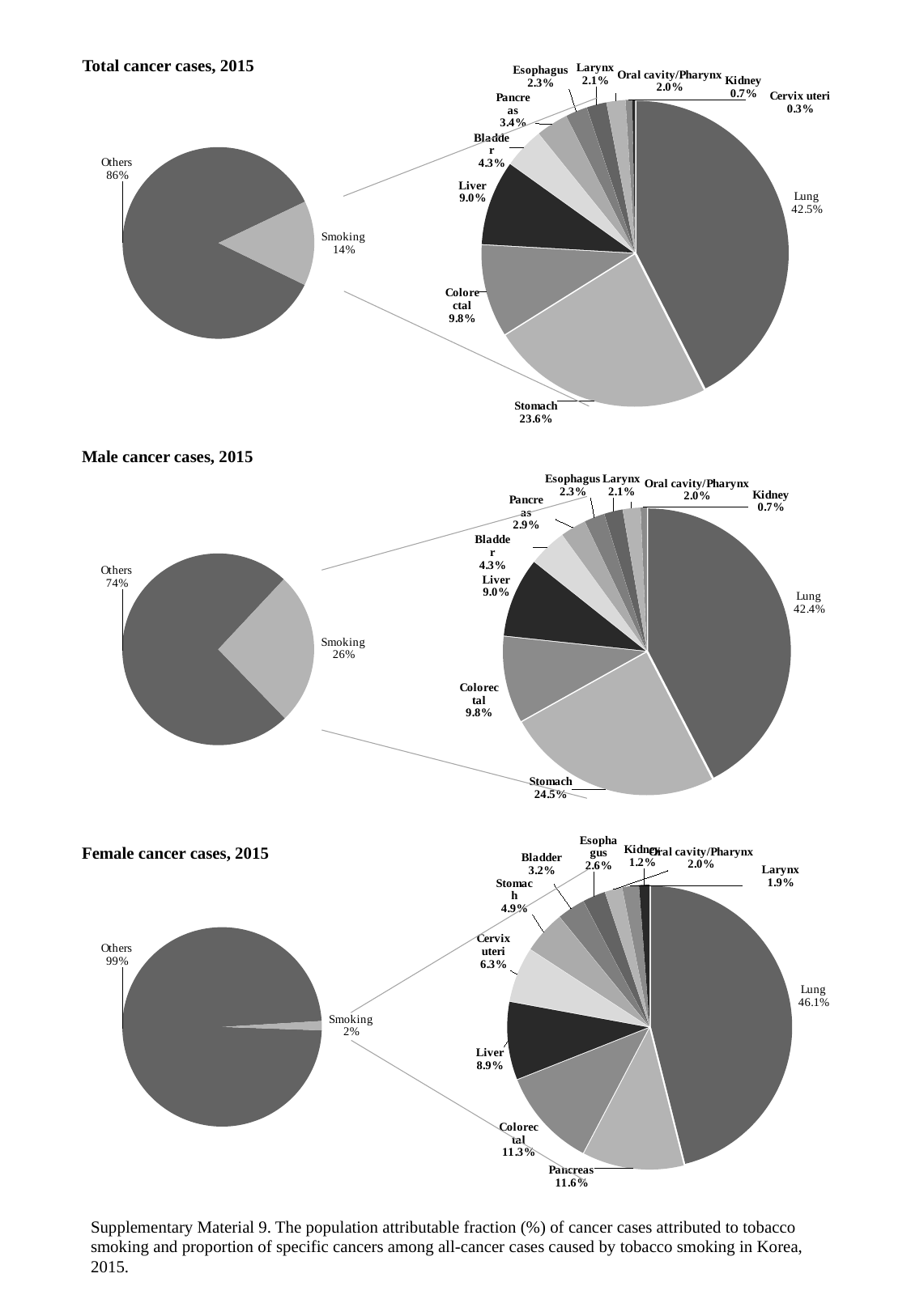

Total cancer cases, 2015
### Chart
| Category | |
|---|---|
| Lung | 42.5 |
| Stomach | 23.6 |
| Colorectal | 9.8 |
| Liver | 9.0 |
| Bladder | 4.3 |
| Pancreas | 3.4 |
| Esophagus | 2.3 |
| Larynx | 2.1 |
| Oral cavity/Pharynx | 2.0 |
| Kidney | 0.7 |
| Cervix uteri | 0.3 |
### Chart
| Category | |
|---|---|
| Others | 85.7 |
| Smoking | 14.3 |Male cancer cases, 2015
### Chart
| Category | |
|---|---|
| Lung | 42.4 |
| Stomach | 24.5 |
| Colorectal | 9.8 |
| Liver | 9.0 |
| Bladder | 4.3 |
| Pancreas | 2.9 |
| Esophagus | 2.3 |
| Larynx | 2.1 |
| Oral cavity/Pharynx | 2.0 |
| Kidney | 0.7 |
### Chart
| Category | |
|---|---|
| Others | 74.2 |
| Smoking | 25.8 |
### Chart
| Category | |
|---|---|
| Lung | 46.1 |
| Pancreas | 11.6 |
| Colorectal | 11.3 |
| Liver | 8.9 |
| Cervix uteri | 6.3 |
| Stomach | 4.9 |
| Bladder | 3.2 |
| Esophagus | 2.6 |
| Oral cavity/Pharynx | 2.0 |
| Larynx | 1.9 |
| Kidney | 1.2 |
### Chart
| Category | |
|---|---|
| Others | 98.5 |
| Smoking | 1.5 |Female cancer cases, 2015
Supplementary Material 9. The population attributable fraction (%) of cancer cases attributed to tobacco smoking and proportion of specific cancers among all-cancer cases caused by tobacco smoking in Korea, 2015.
