# Supplementary material for: Preventable cancer cases and deaths attributable to tobacco smoking in Korea from 2015 to 2030
Source: Epidemiol Health. 2025 Feb 27;47:e2025008. doi: 10.4178/epih.e2025008 (PMC12531467; doi:10.4178/epih.e2025008)
Supplement: Supplementary Material 10. — The population attributable fraction (%) of cancer deaths attributed to tobacco smoking and proportion of specific cancers among all-cancer deaths caused by tobacco smoking in Korea, 2015. [file epih-47-e2025008-Supplementary-10.pptx]

## Slide 1
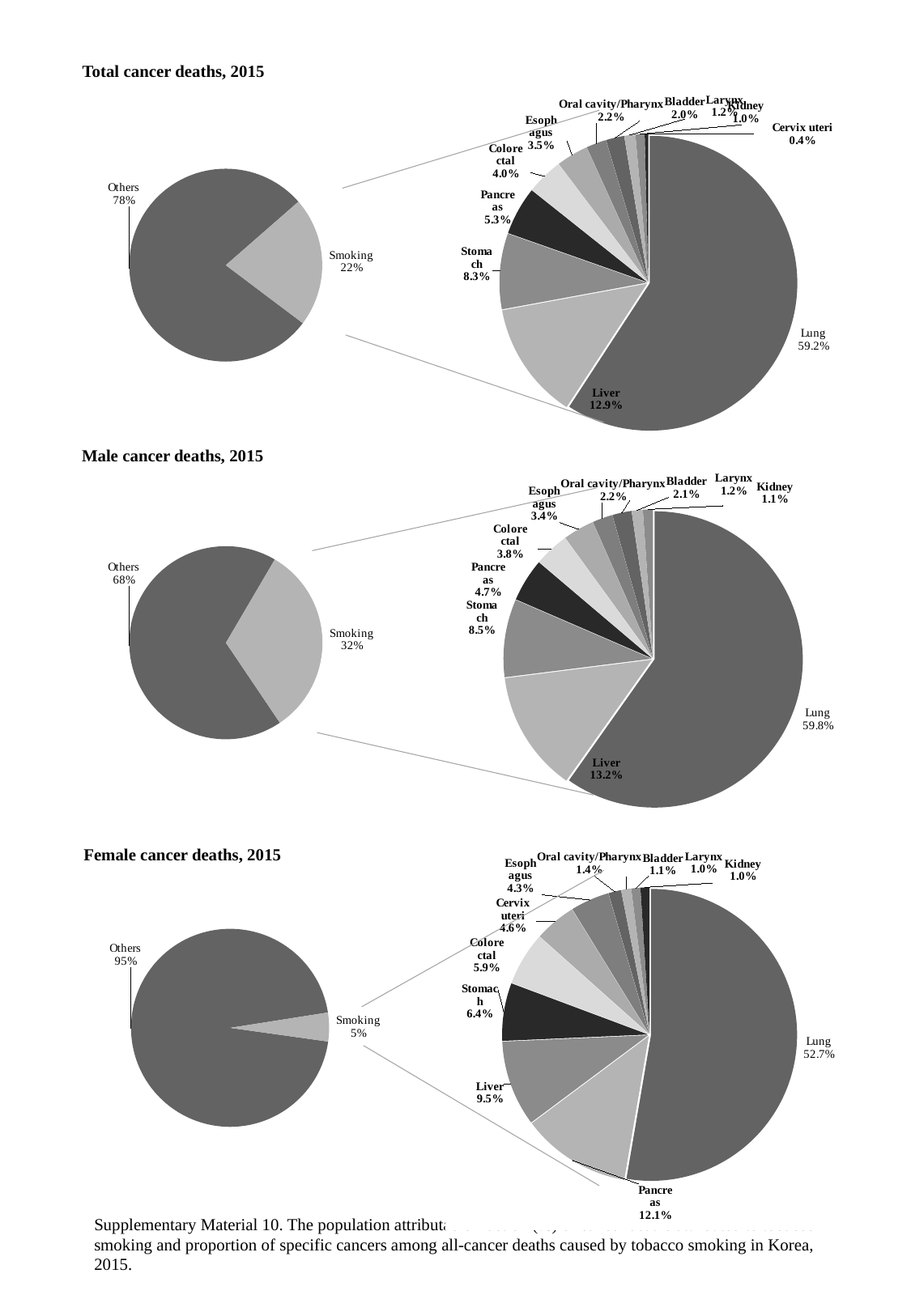

Total cancer deaths, 2015
### Chart
| Category | |
|---|---|
| Lung | 59.2 |
| Liver | 12.9 |
| Stomach | 8.3 |
| Pancreas | 5.3 |
| Colorectal | 4.0 |
| Esophagus | 3.5 |
| Oral cavity/Pharynx | 2.2 |
| Bladder | 2.0 |
| Larynx | 1.2 |
| Kidney | 1.0 |
| Cervix uteri | 0.4 |
### Chart
| Category | |
|---|---|
| Others | 78.3 |
| Smoking | 21.7 |Male cancer deaths, 2015
### Chart
| Category | |
|---|---|
| Lung | 59.8 |
| Liver | 13.2 |
| Stomach | 8.5 |
| Pancreas | 4.7 |
| Colorectal | 3.8 |
| Esophagus | 3.4 |
| Oral cavity/Pharynx | 2.2 |
| Bladder | 2.1 |
| Larynx | 1.2 |
| Kidney | 1.1 |
### Chart
| Category | |
|---|---|
| Others | 67.9 |
| Smoking | 32.1 |
### Chart
| Category | |
|---|---|
| Lung | 52.7 |
| Pancreas | 12.1 |
| Liver | 9.5 |
| Stomach | 6.4 |
| Colorectal | 5.9 |
| Cervix uteri | 4.6 |
| Esophagus | 4.3 |
| Oral cavity/Pharynx | 1.4 |
| Bladder | 1.1 |
| Larynx | 1.0 |
| Kidney | 1.0 |
### Chart
| Category | |
|---|---|
| Others | 95.3 |
| Smoking | 4.7 |Female cancer deaths, 2015
Supplementary Material 10. The population attributable fraction (%) of cancer deaths attributed to tobacco smoking and proportion of specific cancers among all-cancer deaths caused by tobacco smoking in Korea, 2015.
