# Supplementary material for: Preventable cancer cases and deaths attributable to tobacco smoking in Korea from 2015 to 2030
Source: Epidemiol Health. 2025 Feb 27;47:e2025008. doi: 10.4178/epih.e2025008 (PMC12531467; doi:10.4178/epih.e2025008)
Supplement: Supplementary Material 11. — Comparison of population attributable fraction (PAF, %) in specific cancer attributed to tobacco smoking when using different relative risks (RRs). [file epih-47-e2025008-Supplementary-11.pptx]

## Slide 1
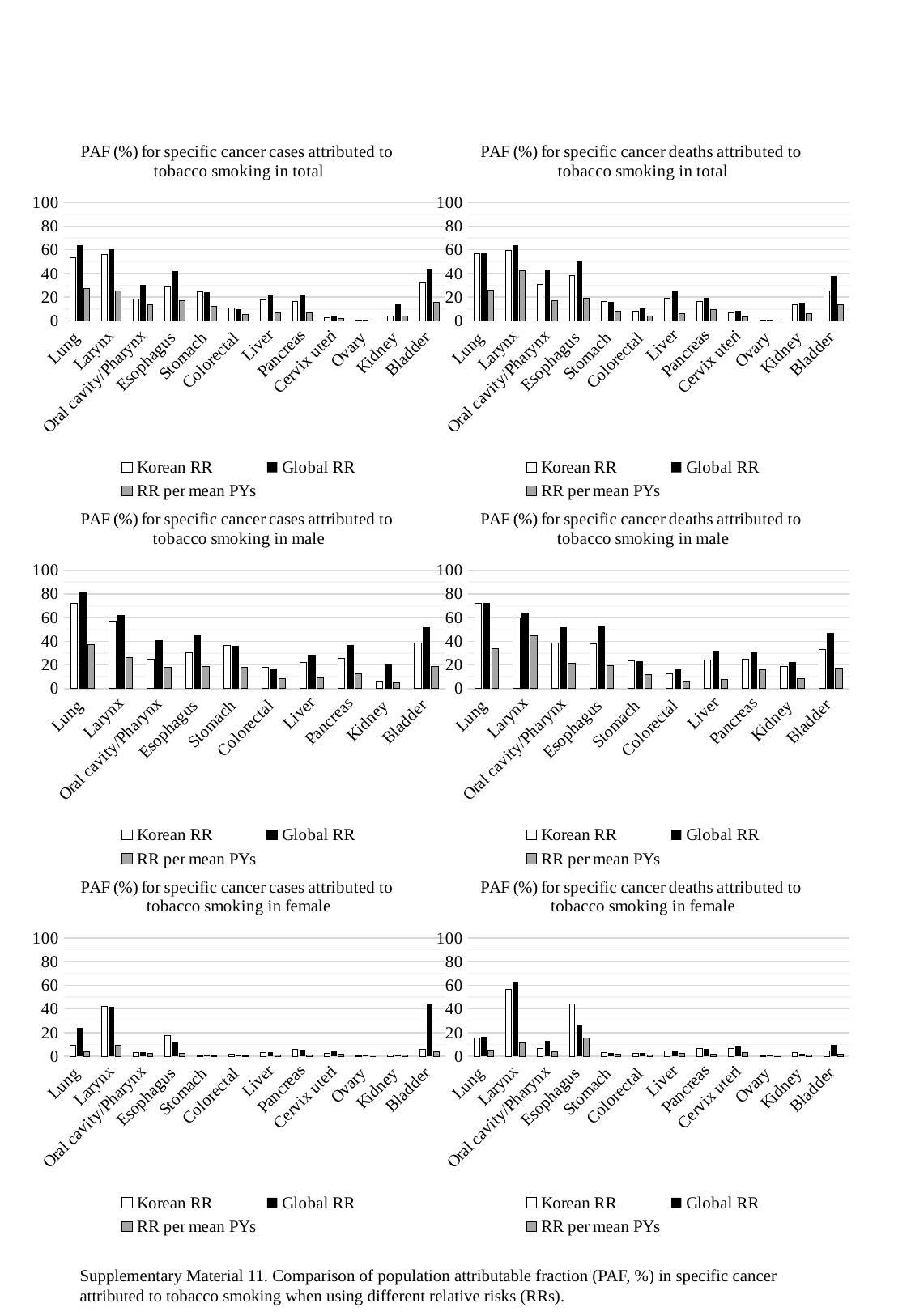

### Chart: PAF (%) for specific cancer cases attributed to tobacco smoking in total
| Category | Korean RR | Global RR | RR per mean PYs |
|---|---|---|---|
| Lung | 53.36 | 63.69 | 27.19 |
| Larynx | 55.88 | 60.78 | 25.06 |
| Oral cavity/Pharynx | 18.64 | 30.43 | 13.86 |
| Esophagus | 29.3 | 42.16 | 16.95 |
| Stomach | 24.75 | 24.55 | 12.23 |
| Colorectal | 11.19 | 10.17 | 5.33 |
| Liver | 17.44 | 21.77 | 6.87 |
| Pancreas | 16.27 | 22.24 | 7.12 |
| Cervix uteri | 2.54 | 4.26 | 1.66 |
| Ovary | 0.51 | 0.88 | 0.09 |
| Kidney | 4.0 | 14.0 | 3.87 |
| Bladder | 32.0 | 44.23 | 15.87 |
### Chart: PAF (%) for specific cancer deaths attributed to tobacco smoking in total
| Category | Korean RR | Global RR | RR per mean PYs |
|---|---|---|---|
| Lung | 56.53 | 57.57 | 26.17 |
| Larynx | 59.73 | 63.92 | 42.18 |
| Oral cavity/Pharynx | 30.69 | 42.91 | 16.96 |
| Esophagus | 38.2 | 50.32 | 18.82 |
| Stomach | 16.16 | 16.24 | 8.06 |
| Colorectal | 8.03 | 10.46 | 3.76 |
| Liver | 18.94 | 24.79 | 6.09 |
| Pancreas | 16.13 | 19.19 | 9.34 |
| Cervix uteri | 6.47 | 8.24 | 3.05 |
| Ovary | 0.51 | 0.88 | 0.09 |
| Kidney | 13.58 | 15.58 | 6.31 |
| Bladder | 25.31 | 38.02 | 13.57 |
### Chart: PAF (%) for specific cancer cases attributed to tobacco smoking in male
| Category | Korean RR | Global RR | RR per mean PYs |
|---|---|---|---|
| Lung | 72.27 | 80.55 | 37.31 |
| Larynx | 56.73 | 61.94 | 26.04 |
| Oral cavity/Pharynx | 24.5 | 40.62 | 18.14 |
| Esophagus | 30.47 | 45.12 | 18.35 |
| Stomach | 36.58 | 36.05 | 18.08 |
| Colorectal | 17.81 | 16.37 | 8.5 |
| Liver | 22.32 | 28.34 | 8.79 |
| Pancreas | 25.67 | 36.76 | 12.4 |
| Kidney | 5.41 | 19.79 | 5.23 |
| Bladder | 38.4 | 51.75 | 18.79 |
### Chart: PAF (%) for specific cancer deaths attributed to tobacco smoking in male
| Category | Korean RR | Global RR | RR per mean PYs |
|---|---|---|---|
| Lung | 71.92 | 72.13 | 34.03 |
| Larynx | 59.96 | 64.01 | 44.58 |
| Oral cavity/Pharynx | 38.41 | 51.84 | 21.25 |
| Esophagus | 37.62 | 52.52 | 19.12 |
| Stomach | 23.43 | 23.43 | 11.66 |
| Colorectal | 12.48 | 16.28 | 5.82 |
| Liver | 24.02 | 32.06 | 7.47 |
| Pancreas | 24.5 | 30.38 | 15.95 |
| Kidney | 18.34 | 22.77 | 8.62 |
| Bladder | 32.72 | 47.35 | 17.65 |
### Chart: PAF (%) for specific cancer cases attributed to tobacco smoking in female
| Category | Korean RR | Global RR | RR per mean PYs |
|---|---|---|---|
| Lung | 9.22 | 24.32 | 3.56 |
| Larynx | 42.01 | 42.01 | 9.1 |
| Oral cavity/Pharynx | 3.21 | 3.63 | 2.58 |
| Esophagus | 17.41 | 12.1 | 2.64 |
| Stomach | 0.75 | 1.19 | 0.35 |
| Colorectal | 1.51 | 1.11 | 0.69 |
| Liver | 3.23 | 3.26 | 1.3 |
| Pancreas | 5.68 | 5.89 | 1.19 |
| Cervix uteri | 2.54 | 4.26 | 1.66 |
| Ovary | 0.51 | 0.88 | 0.09 |
| Kidney | 0.97 | 1.54 | 0.95 |
| Bladder | 5.89 | 43.97 | 3.97 |
### Chart: PAF (%) for specific cancer deaths attributed to tobacco smoking in female
| Category | Korean RR | Global RR | RR per mean PYs |
|---|---|---|---|
| Lung | 15.19 | 16.67 | 5.07 |
| Larynx | 56.71 | 63.06 | 11.63 |
| Oral cavity/Pharynx | 6.84 | 13.26 | 3.71 |
| Esophagus | 44.48 | 26.02 | 15.52 |
| Stomach | 2.89 | 2.89 | 1.5 |
| Colorectal | 2.22 | 2.9 | 1.07 |
| Liver | 4.4 | 4.59 | 2.14 |
| Pancreas | 6.51 | 6.31 | 1.74 |
| Cervix uteri | 6.47 | 8.24 | 3.05 |
| Ovary | 0.51 | 0.88 | 0.09 |
| Kidney | 3.14 | 2.19 | 1.25 |
| Bladder | 4.34 | 9.58 | 2.02 |Supplementary Material 11. Comparison of population attributable fraction (PAF, %) in specific cancer attributed to tobacco smoking when using different relative risks (RRs).
