# Supplementary material for: Preventable cancer cases and deaths attributable to tobacco smoking in Korea from 2015 to 2030
Source: Epidemiol Health. 2025 Feb 27;47:e2025008. doi: 10.4178/epih.e2025008 (PMC12531467; doi:10.4178/epih.e2025008)
Supplement: Supplementary Material 12. — Comparison between cancer PAFs1 caused by tobacco2 in 2009 and 2015 [file epih-47-e2025008-Supplementary-12.docx]

Supplementary Material 12. Comparison between cancer PAFs^1^ caused by tobacco^2^ in 2009 and 2015

|  | **Cancer incidence** | | | | **Cancer death** | | | |
| --- | --- | --- | --- | --- | --- | --- | --- | --- |
|  | **2009** | | **2015** | | **2009** | | **2015** | |
|  | **PAF (%)** | **AC** | **PAF (%)** | **AC** | **PAF (%)** | **AC** | **PAF (%)** | **AC** |
| **Total population** |  |  |  |  |  |  |  |  |
| Lung (C33-C34) | 39.85 | 7,522 | 53.36 | 13,127 | 54.37 | 8,110 | 56.53 | 9,834 |
| Larynx (C32) | 70.25 | 798 | 55.88 | 645 | 65.80 | 279 | 59.73 | 205 |
| Oral cavity (C00-C09) | 37.20 | 610 | 18.64^2^ | 617^2^ | 47.09 | 259 | 30.69^2^ | 357^2^ |
| Pharynx (C10-C14) | 42.01 | 342 |  |  | 55.08 | 233 |  |  |
| Esophagus (C15) | 43.97 | 922 | 29.30 | 716 | 50.57 | 711 | 38.20 | 585 |
| Stomach (C16) | 19.43 | 5,707 | 24.75 | 7,275 | 20.85 | 2,112 | 16.16 | 1,377 |
| Colorectal (C18-C20) | 0.91 | 226 | 11.19 | 3,035 | 0.82 | 58 | 8.03 | 666 |
| Liver (C22) | 19.01 | 2,950 | 17.44 | 2,772 | 19.12 | 2,148 | 18.94 | 2,142 |
| Pancreas (C25) | 15.53 | 661 | 16.27 | 1,038 | 14.91 | 605 | 16.13 | 878 |
| Cervix uteri (C53) | 4.04 | 68 | 2.54 | 92 | 3.99 | 34 | 6.47 | 63 |
| Ovary (C56) | 0.51 | 19 | 0.51 | 12 | 3.05 | 30 | 0.51 | 5 |
| Kidney (C64-C66) | 5.21 | 175 | 4.00 | 225 | 5.14 | 38 | 13.58 | 182 |
| Bladder (C67) | 35.35 | 1,110 | 32.00 | 1,316 | 34.02 | 328 | 25.31 | 329 |
| **All cancer** | **11.90** | **22,368** | **14.32** | **30,870** | **22.83** | **15,848** | **21.70** | **16,623** |
| **Male** |  |  |  |  |  |  |  |  |
| Lung (C33-C34) | 53.34 | 7,244 | 72.27 | 12,447 | 71.45 | 7,783 | 71.92 | 9,117 |
| Larynx (C32) | 72.99 | 782 | 56.73 | 617 | 71.91 | 275 | 59.96 | 191 |
| Oral cavity (C00-C09) | 45.83 | 517 | 24.50^2^ | 588^2^ | 61.95 | 246 | 38.41^2^ | 338^2^ |
| Pharynx (C10-C14) | 45.83 | 322 |  |  | 61.95 | 228 |  |  |
| Esophagus (C15) | 47.21 | 919 | 30.47 | 678 | 54.83 | 711 | 37.62 | 527 |
| Stomach (C16) | 27.89 | 5,514 | 36.58 | 7,202 | 31.56 | 2,107 | 23.43 | 1,290 |
| Colorectal (C18-C20) | 1.50 | 224 | 17.81 | 2,868 | 1.16 | 45 | 12.48 | 586 |
| Liver (C22) | 23.47 | 2,737 | 22.32 | 2,640 | 23.47 | 1,976 | 24.02 | 2,013 |
| Pancreas (C25) | 27.40 | 646 | 25.67 | 868 | 26.84 | 598 | 24.50 | 713 |
| Kidney (C64-C66) | 7.62 | 175 | 5.41 | 208 | 6.61 | 33 | 18.34 | 169 |
| Bladder (C67) | 43.39 | 1,093 | 38.40 | 1,268 | 44.87 | 318 | 32.72 | 314 |
| **All cancer** | **20.94** | **20,271** | **25.83** | **29,384** | **32.91** | **14,368** | **32.09** | **15,258** |
| **Female** |  |  |  |  |  |  |  |  |
| Lung (C33-C34) | 5.25 | 278 | 9.22 | 680 | 8.13 | 327 | 15.19 | 717 |
| Larynx (C32) | 24.01 | 16 | 42.01 | 28 | 9.21 | 4 | 56.71 | 14 |
| Oral cavity (C00-C09) | 18.19 | 93 | 3.21^2^ | 29^2^ | 8.23 | 13 | 6.84^2^ | 19^2^ |
| Pharynx (C10-C14) | 18.19 | 20 |  |  | 8.23 | 5 |  |  |
| Esophagus (C15) | 2.32 | 3 | 17.41 | 38 | 0.18 | 0 | 44.48 | 58 |
| Stomach (C16) | 2.01 | 193 | 0.75 | 73 | 0.16 | 5 | 2.89 | 87 |
| Colorectal (C18-C20) | 0.02 | 2 | 1.51 | 167 | 0.42 | 13 | 2.22 | 80 |
| Liver (C22) | 5.53 | 213 | 3.23 | 132 | 6.11 | 172 | 4.40 | 129 |
| Pancreas (C25) | 0.77 | 15 | 5.68 | 170 | 0.39 | 7 | 6.51 | 165 |
| Cervix uteri (C53) | 4.04 | 68 | 2.54 | 92 | 4.04 | 34 | 6.47 | 63 |
| Ovary (C56) | 0.51 | 19 | 0.51 | 12 | 3.08 | 30 | 0.51 | 5 |
| Kidney (C64-C66) | 0.03 | 0 | 0.97 | 17 | 2.29 | 5 | 3.14 | 13 |
| Bladder (C67) | 2.77 | 17 | 5.89 | 48 | 3.75 | 10 | 4.34 | 15 |
| **All cancer** | **2.30** | **2,097** | **1.46** | **1,486** | **5.74** | **1,480** | **4.70** | **1,365** |

Abbreviation: PAF, Population attributable fraction; AC, Number of attributable cancer cases or deaths.

1. PAF in 2015 was calculated with 15 years of latency, and PAF in 2009 was calculated with 19 years of latency.

2. Tobacco smoking was classified to current smoking, past smoking, and never smoking (reference).
